# Supplementary material for: Continuous and scalable manufacture of amphibious energy yarns and textiles
Source: Nat Commun. 2019 Feb 20;10:868. doi: 10.1038/s41467-019-08846-2 (PMC6382889; doi:10.1038/s41467-019-08846-2)
Supplement: Supplementary file 1 — Supplementary Information [file 41467_2019_8846_MOESM1_ESM.pdf]

Supplementary Information

Continuous and scalable manufacture of amphibious energy yarns and textiles

Wei Gong[1], Chengyi Hou[1]*, Jie Zhou[3], Yinben Guo[1], Wei Zhang[1], Yaogang Li[2], Qinghong Zhang[2]*, Hongzhi Wang[1]*

[1]State Key Laboratory for Modification of Chemical Fibers and Polymer Materials, College of Materials Science and Engineering, Donghua University, Shanghai 201620, P. R. China

[2]Engineering Research Center of Advanced Glasses Manufacturing Technology, Ministry of Education, Donghua University, Shanghai 201620, P. R. China

[3]College of Electronics and Information Engineering, Sichuan University, Chengdu 610064, P. R. China

*Corresponding author: Chengyi Hou (hcy@dhu.edu.cn), Qinghong Zhang (zhangqh@dhu.edu.cn), Hongzhi Wang (wanghz@dhu.edu.cn)

Supplementary Notes

Supplementary Note 1

As illustrated in Figure 1b, a stainless steel yarn is pulled along the equipment by the compaction roller II (Supplementary Figure 1c), while the silicone rubber masterbatch is poured into the extruder (Supplementary Figure 1a) to melt at 250–280 ° C. Subsequently, the melted silicone rubber enters area II where it transforms to pre-formed state at 120–180 °C. In area III (30-50 ° C), silicone rubber tube is evenly obtained through blow-molding. Silicone rubber is evenly expanded around due to the expansion of the frozen compressed gas (compressed gas pressure about 0.3-0.7 MPa) and the temperature of silicone rubber is drastically reduced at the same time, thus the formed silicone rubber tube is obtained. It is stretched forward by the traction force from the compaction roller I, and a stainless steel yarn is stretched forward by the traction force from the compaction roller II (Figure 1a). From the cross-section enlarged view of compaction roller I, it can be seen that the compaction roller I only acts on the silicone rubber tube without stretching the stainless steel yarn. Note that in areas I and II, the frozen compressed gas does not affect the melting and pre-forming process due to the barrier effect of thermal insulation. In addition, the entire molding process is carried out in a hollow equipment with the hollow interlayer (Supplementary Figure 2). The silicone rubber moves in the hollow interlayer, and the stainless steel yarn is always in the center of hollow device and is moved forward by the traction force.

Supplementary Note 2

For silicone rubber tubes, the difference in size during stretching will only change the value of the tensile force without changing the overall trend of the tensile force. Therefore, according to the fitted line of Supplementary Figure 3, it can be reasonably assumed that there is a relationship between tensile force (F_{x1}) and pre-strain (ε) as follows:

$$F_{x1} = m\varepsilon + b \qquad (1)$$

where m and b are the slope and intercept of the fitted line, respectively. In addition, the resultant gravity (F_y) and tensile force (F_{x1}) have the following relationship:

$$F_{x1} = \frac{F_y}{n} \qquad (2)$$

where n is the number of pulley blocks.

Supplementary Note 3

In the stress releasing process, the tensile force (F_{x2}) of silicone rubber tube is always less than F_{x1}. The rotational speed relationship between compaction roller II and reeling roller can be expressed by the following formula:

$$2\pi r_2 V_2 = (1+\varepsilon)2\pi r_3 V_3 \quad (3)$$

$$V_3 = \frac{r_2 V_2}{(1+\varepsilon) r_3} \quad (4)$$

where r_2 and r_3 are radius of the compaction roller II and reeling roller, V_2 and V_3 are rotational speed of the compaction roller II and reeling roller, and ε is pre-strain of the silicone rubber tube. V_3 can be effectively determined by r_2, V_2 and r_3 under a fixed ε.

Supplementary Note 4

To explore the mechanical properties of silicone rubber tube and SETEY, we performed tensile tests at different strain rates (10 to 500 mm min^{-1}). For silicone rubber tube, the stress-strain behavior is characterized by three regions depending on the extent of deformation, as shown in Supplementary Figure 4a. In each region, the stress increases nearly linearly with strain, and the rate of increase describes the Young's modulus of the material. After pre-stretching to approximately 200% strain, Supplementary Figure 4a also shows the mechanical properties of SETEY. Interestingly, at small deformation in Region I the Young's modulus shows no obvious change (Supplementary Figure 4b). Supplementary Figure 4c-e present the stress-strain curve of SETEY and silicone rubber tube are stretched 200% during the tensile cycle test under different deformation rates, respectively. Closed curve reveals good elastic deformation of SETEY at a strain of 200%. In summary, the maximum tensile strain of silicone rubber tube can reach ~ 600%, and the elastic deformation of SETEY can reach ~ 200%. Unless otherwise mentioned, a SETEY been pre-stretched to 100% is used in various characterizations as demonstrated below.

Supplementary Note 5

The stress/strain transfer in different materials/structures barely equal under normal conditions. However, there are some abnormal phenomena in our work due to the special material selection and structural design.

On the one hand, as shown in Supplementary Figure 3 and Supplementary Movie 3, the stretching of the silicone rubber tube is a linear elastic deformation obeying Hooke's law when the tensile strain is less than 200%, so the stress/strain on the silicone rubber tube is uniformly transferred. On the other hand, the deformation of the stainless steel helix with extrinsic elasticity is simultaneously affected by the two sealed ends of the triboelectric yarn and the inner wall of the silicone rubber tube. It is worth noting that the stretch length of each point of a helical structure (such as a spring) obeying Hooke's law follows a certain relationship during stretching. As shown in Supplementary Figure 6a, it is assumed that the left end of the helical structure 1 is fixed, and a force is applied on its right end to stretch forward to obtain the helical structure 2. There is the following relationship between the helical structure 1 and the helical structure 2:

$$\mathrm{d}L_{\mathrm{m}} = \frac{L_{\mathrm{m}}}{L_{\mathrm{n}}}\mathrm{d}L_{\mathrm{n}} \qquad (5)$$

where L_{n} is the distance from point n in the helical structure 1 to the left end of the helical structure 1 (i.e., the length of the helical structure 1), and L_{m} is the distance from point m in the helical structure 1 to the left end of the helical structure 1. $\mathrm{d}L_{\mathrm{n}}$ and $\mathrm{d}L_{\mathrm{m}}$ are stretching distance of points n and m, respectively, when helical structure 1 is stretched to helical structure 2. In Supplementary Figures 6b and 6c, the marks a and b on the triboelectric yarn are stretched to marks A and B, respectively, after 2.792 s. In Supplementary Figure 6b, the distance from mark a to the left end of the triboelectric yarn is approximately half the distance from the mark b to the left end. From the scale in the Supplementary Figure 6c, it can be seen that the stretching distance of b is exactly twice the stretching distance of a. Therefore, the stainless steel helix also obeys Hooke's law during the operation of the triboelectric yarn. In addition, as shown in Supplementary Figure 7, the stainless steel yarn and the silicone rubber tube are marked with y and t, respectively. The stainless steel yarn and the silicone rubber tube are always synchronously deformed during triboelectric yarn stretching (y1 to y4, t1 to t4). And Supplementary Figure 4 further confirms that the triboelectric yarn has good mechanical cycle stability.

Supplementary Note 6

To further verify the stability of helical structure, a 30 cm SETEY is cut and the helical yarn maintains its shape at the fracture (Supplementary Figure 8a-c). This is owing to the SETEY mainly depends on the friction between the silicone rubber tube wall and the stainless steel yarn to maintain the shape of the helical yarn. The remainder of the SETEY is sealed with scotch tape at the fracture, then we test its output

performance. The voltages of remainders (remaining ratios are 100%, 90%, 80%, 50%, 10%, respectively) are exhibited in Supplementary Figure 8d, and it decreases with the decrease of the remaining ratio. Therefore, the SETEY still has good output performance when it is sealed again after shearing.

Supplementary Note 7

Working principle of the SETEY. The SETEY is a single-electrode triboelectric nanogenerator. Working principle of the SETEY is schematically depicted by the coupling of contact electrification and electrostatic induction (Supplementary Figure 9). In order to better understand the working mechanism of SETEY, we present its full working cycle through the front view (Supplementary Figure 9a_1-d_1) and the side view (Supplementary Figure 9a_2-d_2), respectively. It is notable that the number of positive and negative charges is always equal in the front view and the sectional drawing. However, in the schematic diagram, partial charges are hidden due to differences in viewing angles and are not presented. In the entire system, all negative charges are located on the internal surface of the silicone rubber tube, and all positive charges are located on the stainless steel yarn and the ground electrode.

In the original position, the internal surface of silicone rubber tube and the helical stainless steel yarn fully contact each other, resulting in charge transfer between them. According to the triboelectric series [1], electrons are injected from the stainless steel yarn to the silicone rubber tube since silicone rubber tube is more triboelectrically negative than stainless steel yarn, which is the contact electrification process. The produced negative triboelectric charges can be preserved on the internal surface of the silicone rubber tube for a long time due to the nature of the insulator [2]. The produced triboelectric charges with opposite polarities are fully screened, leading to no electron flow in the external circuit. Once the silicone rubber tube is stretched, there will be an alteration of the distribution of triboelectric charges. On one hand, the contact area between the silicone rubber tube and the stainless steel yarn is reduced, which causes charge separation. On the other hand, the charge density on the internal surface of the silicone rubber tube will decrease due to the increased internal surface area, so negative charge on the silicone rubber tube within the contact area will decrease. The charge separation and the charge density decrease on the internal surface of the silicone rubber tube will drive electrons to flow from the ground to the stainless steel yarn electrode through the load and finally reach an equilibrium. The transfer of charges between the stainless steel yarn electrode and the ground will continue until the silicone rubber tube stops elongating, where the amount of

transferred charges reach maximum values. When the silicone rubber tube is reverted to move backward, the induced positive charges on the stainless steel yarn increase, driving the electrons to flow from the stainless steel yarn to the ground to produce a current signal. Finally, when the silicone rubber tube gets back to its original state, the charged surfaces contact again and there will be no change of the induced charges on the stainless steel yarn, thus no output current can be observed. This is a full cycle of the SETEY working process. Here, the outer silicone rubber tube can be used as a dielectric layer due to its well attract electrons ability, and it can also be used as a protection layer of generator because of its hydrophobicity. There is no other dielectric layer in this structure, and the inner stainless steel yarn plays two roles: as the triboelectric layer and as the electrode. Therefore, the charges in the inner stainless steel yarn have two parts: one is the triboelectric charges, the other is the transferred charges between the inner stainless steel electrode and ground electrode.

Supplementary Note 8

The constructed model is based on the factors that stainless steel yarn has a diameter of 0.45 mm and length of 60 cm, and the silicone rubber tube has the dimensions of 0.8 mm × 1.8 mm × 30 cm (inner diameter, outer diameter and length). The triboelectric charge on the silicone rubber tube was assumed to be 36 nC. Note that the amount of initial triboelectric charges will only affect the magnitudes of the calculated potential, but the relative changing of the electrical signals will remain constant [3,4]. The stainless steel yarn was connected with the ground. Figure 2c depicts the calculated results of the electric potential distribution in the TENG when the silicone rubber tube is stretched to various magnitudes (0, 10 cm, 20 cm, and 30 cm). When the internal surface of the silicone rubber tube and the helical stainless steel yarn fully contact each other, the electric potential on the internal surface of the silicone rubber tube approaches zero. When the silicone rubber tube is stretched 30 cm, the electric potential on the internal surface of the silicone rubber tube is up to -1200 V. However, the electric potential on the stainless steel yarn is still close to zero because of the external electron injection. It can be clearly seen that the electric potential difference between the internal surface of the silicone rubber tube and the stainless steel yarn increases dramatically with increasing tensile length.

Supplementary Note 9

In order to achieve excellent electrical outputs, the structure and working mode should be optimized. The mechanism of the SETEY operation was presented in Figure 2, Supplementary Notes 7 and 8. It can be known that the factor that really affects the performance of SETEY is the contact area and separation distance between the silicone rubber tube and the stainless steel yarn at the same working frequency.

On contact area factor: the contact area is determined by the diameter of the stainless steel yarn, the inner diameter of the silicone rubber tube, and the length of the SETEY.

On separation distance factor: the separation distance is determined by the diameter of the stainless steel yarn, the inner diameter of the silicone rubber tube, and the tensile strain.

Therefore, the output performance of the SETEY is determined by the diameter of the stainless steel yarn, the inner diameter of the silicone rubber tube, the length of the SETEY, and the tensile strain.

When the silicone rubber tube is stretched outward, its length increases and the inner diameter becomes smaller, as schematically shown in Supplementary Figure 10a. Since the internal volume of the silicone rubber tube is always constant before and after stretching, the inner diameter of the silicone rubber tube has the following expression:

$$\pi\left(\frac{D_0}{2}\right)^2 L_0 = \pi\left(\frac{D_s}{2}\right)^2 L_s \qquad (6)$$

$$D_s = D_0\sqrt{\frac{L_0}{L_s}} \qquad (7)$$

where D_0 is the inner diameter of the silicone rubber tube in the original state, L_0 is the length of the silicone rubber tube in original state, D_s is the inner diameter of the silicone rubber tube in the pre-stretching state, and L_s is the length of the silicone rubber tube in pre-stretching state. Furthermore, we find that the inner diameter of the silicone rubber tube and its pre-strain have the following relationship:

$$\varepsilon = \frac{L_s - L_0}{L_0} \qquad (8)$$

$$D_s = D_0\sqrt{\frac{1}{1+\varepsilon}} \qquad (9)$$

where ε is pre-strain of the silicone rubber tube. Note that regardless of how the inner diameter of the silicone rubber tube changes, it is always larger than the diameter of the stainless steel yarn during the stretching of the silicone rubber tube. When the pre-strain (ε) is 100%, the separation distance between the silicone rubber tube and the stainless steel yarn at a tensile strain (ε_t) of 100% can be expressed as:

$$d_{(\varepsilon=100\%)} = \frac{D_s - D_y}{2} \qquad (10)$$

$$d_{(\varepsilon=100\%)} = \frac{1}{2}\left(\frac{D_0}{\sqrt{2}} - D_y\right) \qquad (11)$$

where d is separation distance between the silicone rubber tube and the stainless steel yarn, and D_y is diameter of the stainless steel yarn. According to Equation 11, we can get the separation distance between the silicone rubber tube and the stainless steel yarn under different parameters, as shown in Supplementary Figure 10d and g.

Since the contact area between the silicone rubber tube and the stainless steel yarn is difficult to measure, we roughly estimate the contact area by the number of helices. The number of helices under different parameters are shown in Supplementary Figure 10d and g. The relationship between the output voltage of SETEY and the diameter of the stainless steel yarn is shown in Supplementary Figure 10e.

In Supplementary Figure 10b and 11, it can be found that the output voltage of SETEY first increases with increasing inner diameter, then decreases when the inner diameter exceeds 1.5 mm. Besides, the tensile force also increases with the increase of inner diameter. In Supplementary Figure 10e, the overall output voltage of SETEY decreases as the diameter of the stainless steel yarn increases. However, when the diameter of the stainless steel yarn is less than 0.45 mm, the output performance is only slightly changed. When the diameter of the stainless steel yarn is larger than 0.45 mm, the output performance drops dramatically. However, the 0.45-mm stainless steel yarn has greater breaking strength at this time (Supplementary Figure 10h), so we chose a 0.45-mm stainless steel yarn as the core yarn of SETEY.

Above phenomena are attributed to the variation of contact area and separation distance between core and sheath materials. It is known that the performance of single-electrode mode TENG is greatly affected by the separation distance when the separation distance is small. However, this effect will gradually decrease as the separation distance increases [5]. Here, according to Equation 11, Supplementary Figures 10b, d, e and g, we know that the performance is mainly affected by the separation distance when the separation distance is less than 0.305 mm (D_0 = 1.5 mm and D_y = 0.45 mm). When the separation distance is greater than 0.305 mm, the influence of the separation distance is weakened, and the performance is mainly affected by the contact area.

Similarly, when the length of SETEY increases, the contact area increases and the separation distance does not change. The performance of SETEY increases as the contact area increases (Supplementary Figure 10i and 12). In addition, when the tensile strain changes during the operation, the separation distance changes and the contact area does not change. When the tensile strain is less than 100%, the SETEY is in

an incompletely separated state. Therefore, the separation distance is particularly small at this time, and the performance is greatly affected by the separation distance. The separation distance between the silicone rubber tube and the helical stainless steel yarn during the process of separation increases exponentially with increasing tensile strain, as displayed in Supplementary Figure 17 and 18. Supplementary Figure 10j and 13 show the voltage and the tensile strain can be fitted with exponential curve, with a correlative coefficient of 0.976.

In summary, the overall output performance of the SETEY at the same working frequency is determined by the diameter of the stainless steel yarn, the inner diameter of the silicone rubber tube, the length of the SETEY, and the tensile strain. According to the fitting curves of Supplementary Figures 10c, f, i and j, the following relationship can be obtained between them:

$$V = \begin{cases} a_3 D_0^3 + a_2 D_0^2 + a_1 D_0 + a_0, \ (D_y = 0.45mm, L = 30cm, \varepsilon_t = 100\%) \\ b_1 e^{\frac{-D_y}{t}} + b_2, \ (D_0 = 0.8mm, L = 30cm, \varepsilon_t = 100\%) \\ c_1 L + c_2, \ (D_0 = 0.8mm, D_y = 0.45mm, \varepsilon_t = 100\%) \\ \mathrm{d}_1 e^{\frac{\varepsilon_t}{m}} + d_2, \ (D_0 = 0.8mm, D_y = 0.45mm, L = 30cm) \end{cases} \quad (12)$$

where V is the output voltage of the SETEY, L is the length of the SETEY, and ε_t is the tensile strain of the SETEY. a_3, a_2, a_1 and a_0 are the coefficients of the polynomial fit, where they are 0.37, -2.63, 4.96 and 1.93, respectively. b_1, b_2 and t are the coefficients of the single exponential decay fit, where they are -6.56*10^{-13}, 4.53 and -0.0175, respectively. c_1 and c_2 are the coefficients of the linear fit, where they are 0.151 and -0.342, respectively. d_1, d_2 and m are the coefficients of the exponential fit, where they are 0.17, -0.12 and 30, respectively.

We next demonstrate that the SETEY serve as human strain sensors to discriminate and detect the muscle change of different person during arm bending (Supplementary Figure 14). As shown in Supplementary Figure 10k, the output current of SETEY obviously increase when the bending frequency of arm vary from 0.5 to 2 Hz. A detailed analysis of human motion monitoring is shown in Supplementary Figure 15.

To investigate the practical applications of SETEY, the long-term stability/reliability of SETEY is examined for approximately 2000 cycles (frequency, 0. 5 Hz; length, 30 cm; tensile strain, 100%), as exhibited in Supplementary Figure 16. It is found that there was no decrease in output voltage after 2000 cycles. We also present another two samples (sample 2 was fabricated on September 20 and 3 was fabricated on September 27, while sample 1 presented in original manuscript was fabricated on January 14)

in the revised manuscript. The output voltages of the two samples under stretching mode (strains of 10%, 40%, 70% and 100%) were collected and compared with the data from the manuscript (Supplementary Figure 13, denoted as sample 1), as seen in Supplementary Figure 23 below. From the comparison, the three samples exhibited similar output voltage at different stretching strains, although with a slight discrepancy. We think this slight discrepancy may be normal production error and test error, which still indicates that our triboelectric yarns have good reproducibility.

Supplementary Note 10

We may need to point out the difference in underlying mechanism between our built-in helix structure and flat-yarn structure first. In a flat-yarn structure, the core and shell materials should be both stretchable and at least one of them should be conductive. There raises concerns: (1) the resistance change of the conductive material under stretch movement will interfere (the uniformity and readability of) electrical signals, (2) in a flat structure, there is a lack of sufficient transitional state between the two states of full contact and complete separation, resulting in its electrical strain sensitivity being low, and (3) under stretch movement, the flat core and shell deform very similarly, i.e., elongate at almost same tensile strain, therefore few friction can be expected.

In comparison, our built-in helix structure addresses the resistance-variation issue (Supplementary Figure 19b). Besides, the built-in helix structure achieves the effective transition between full contact and complete separation by the change of the helix angle (i.e., the degree of the helix) (Supplementary Figure 17a).

In addition, based on the same materials and similar fabrication method reported in this work, we fabricate a core-shell yarn-like TENG with a flat core yarn. Its structure and mechanism are illustrated in Supplementary Figure 19a. As shown in Supplementary Figure 19c, it is obvious that the built-in helix structure has a larger and more stable output voltage than the flat structure. The reasons are: (1) the silicone rubber tube has a larger contact area with the stainless steel yarn in the built-in helix structure, which causes it to have a larger output voltage than the flat structure. (2) the built-in helix structure obtains a fixed number of helices by pre-stretching, thereby maintaining a stable contact-separation area; however, the contact area of the flat structure after each stretching may be somewhat deviated, resulting in that the output is not stable.

Supplementary Note 11

By using the Charges and Fields software to analyze the field intensity at different locations, we found that the closer the distance to the point charge group, the greater the field intensity (Supplementary Figure 24). This is owing to the electric field intensity at different locations with its distance to the point charge group has the following relationship.

$$\overrightarrow{\mathbf{E_n}} = q_n \overrightarrow{\mathbf{r_n}} / 4\pi K_s {r_n}^2 \quad (13)$$

$$\vec{\mathbf{E}} = \sum_n \overrightarrow{\mathbf{E_n}} = \frac{1}{4\pi K_s} \sum_n \frac{q_n}{r_n^2} \overrightarrow{\mathbf{r_n}} \quad (14)$$

where $\overrightarrow{\mathbf{E_n}}$ is the electric field intensity of a point charge at a certain point, which is a vector. q_n is the charge of a point charge. $\overrightarrow{\mathbf{r_n}}$ is the radial vector from a certain point in the electric field to a point charge. K_s is the relative dielectric constant of the silicone rubber. $\vec{\mathbf{E}}$ is the total electric field intensity at a certain point under the point charge group. The experimental results in Figure 3b are consistent with this superposition theory of electric field intensity, namely electric field intensity decreases with the increase of the tube wall's thickness. This is expected since the thicker the dielectric layer, the more difficult it is to polarize liquid molecules.

Supplementary Note 12

In Supplementary Figure 25, we summarize the working mechanism of SETEY under water. When the silicone rubber tube and the stainless steel yarn are in contact, they will acquire net opposite charges on their surfaces. Once the silicone rubber tube is stretched, electrons flow from the ground to the stainless steel yarn. Meanwhile, the surface potential of silicone rubber tube polarizes surrounding water molecules. The polarization of water molecules will keep growing until the silicone rubber tube stops elongating. As the silicone rubber tube is restored, the polarization of water molecules will gradually decrease until the silicone rubber tube returns to its original shape. Due to dielectric relaxation [6-8], however, the polarization of water molecules will not fully diminish, and the residual polarization of water molecules will act as a negative charge trap to enhance the silicone rubber tube's capability of capturing charges during contact electrification.

Supplementary Note 13

As shown in the Supplementary Figure 26, there is a nonlinear relationship between the relative dielectric constant and the voltage by fitting, which can be expressed by the following formula:

$$V = \frac{abK^{1-c}}{1+bK^{1-c}} \qquad (15)$$

where K is the relative dielectric constant of the liquid and V is the output voltages of the triboelectric yarn. a, b and c are the equilibrium constants under normal temperature and atmospheric pressure, where they are 14, 0.385 and 0.17, respectively. As the relative dielectric constant of the liquid increases, the output voltage of the triboelectric yarn begins to increase rapidly, and then the growth trend slows down, that is, the slope of the fitting curve gradually decreases. The cause of this phenomenon may be complex, and some hidden factors may play particular roles in output changes. We believe that there are two most likely factors. First, the output voltage is positively correlated with the polarity of the liquid molecules as the polarity of the liquid molecules changes. The other is that interfacial liquid and bulk liquid may also exist similar to water. The thickness and nature of the interfacial liquids are inconsistent in different liquids, which may be the main factor leading to the change in the slope of the fitting curve.

Supplementary Note 14

We found that incorporating them with additional yarns would be a more practical and efficient approach. The reasons are as follows. First, from a practical point of view, the deformation of the fabric is usually uniaxial stretching/contraction, and the deformation in the other direction can be ignored (Figures 4c, Supplementary Figure 27a and 27b). So weaving SETEYs in the weft direction meets the needs of practical applications. Second, the output performance can be further improved by the design of the materials when the SETEYs are woven together with other yarns. Third, fashionable textiles can be woven from the SETEYs and colorful yarns, which is especially important in the commercial applications. Fourth, in single-electrode mode, when the unit spacing of two SETEY is small, the electric field from each SETEY will interfere with each other, resulting in a decrease of the output performance for each SETEY [5]. Only when their spacing is large enough, the mutual influence will be minimized. In our TENG fabric, the warp and weft yarns are in close contact (Supplementary Figure 27c), so the performance of the single SETEY is greatly weakened. As shown in Supplementary Figure 28d, the output voltage of B is smaller than that of A at 100% tensile strain due to mutual interference of electric fields. Besides, the weft yarn remains substantially flat when the two SETEYs are woven into a TENG fabric, as shown in Supplementary Figure

27c. The warp yarn cannot be completely stretched due to the barrier of the weft yarn, thereby the output of the warp yarn (C) is smaller than the weft yarn (B), as shown in Supplementary Figure 27d.

Supplementary Note 15

The SETEY can be woven as the same as weaving hair due to good flexibility, so we weave different numbers of SETEYs into one-ply, three-ply, four-ply, five-ply, and six-ply devices (Supplementary Figure 28a). Supplementary Figure 28b displays the circuit diagrams of the voltage and current testing systems after weaving. The results indicate that increasing the number of SETEY leads to an increase in output voltage and current. As shown in Supplementary Figure 28c, for woven SETEY device, the measured values of voltage increase from ~4.5 V to ~10.1 V, and that of current increases from ~0.37 μA to ~0.83 μA as the number of SETEY increasing from 1 to 6. It is clear that the rate of output performance growth is less than the rate of number growth. This can be explained by: firstly, the interwoven structure makes the silicone rubber tube and the helical stainless steel yarn impossible to completely separate during stretching, and second, electric field from each SETEY may interfere with each other because the unit spacing between SETEYs is too small, resulting in a decrease of output performance for each SETEY.

Supplementary Note 16

Ordinary plied yarns are generally formed by twisting parallel fiber bundles. When the twist shringage reach equilibrium, outer layer fibers of the yarn are stretched and inner layer fibers of the yarn are compressed axially. In fact, internal anti-stress of the outer fiber is inward when the outer fiber is stretched, and internal anti-stress of the inner fiber is outward when the inner fiber is compressed (Supplementary Figure 31I). In addition, there is torsional anti-stress around center of the plied yarn, and direction of the torsional anti-stress is direction of the yarn's untwisting (Supplementary Figure 31I). Because the cross-section of the yarn is not a true circle, the center (O_S) of internal anti-stress resultant (F_S) of the outer fiber does not coincide with the center (O_P) of internal anti-stress resultant (F_P) of the inner fiber, and their eccentricity is d (Supplementary Figure 31II and 31III). F_S and F_P are equal in size and opposite in direction, respectively. Their relationship can be expressed by Equation 16:

$$M_0 = F_s d = F_p d \qquad (16)$$

where M_0 is couple moment. Couple moment can bend the yarn so that the yarn exhibits good flexibility. Because stainless steel yarn has excellent flexibility that ordinary wire does not have, we chose it as the core yarn of SETEY. Further, torsional anti-stress can create torsional moments (M_1, M_2) that cause the yarn to rotate, which will greatly reduce the yarn's helix. Therefore, the existence of couple moment is a normal phenomenon of a primary plied yarn, whereas torsional moments (M_1, M_2) are not conducive to maintaining twisting structure of the yarn. So we combine the two primary plied yarns first and then twist them in the opposite direction to before (Supplementary Figure 31IV). The following formula can be obtained:

$$M_3 = M_1 + M_2 \tag{17}$$

where M_3 is torsional moment of the double-plied yarn, and M_1 and M_2 are torsional moments of the two primary plied yarns, respectively. This way can reduce nonuniform tension during twisting and provide comparably highperformance yarns.

Supplementary Note 17

Ideal fabric structure. When the warp and weft yarns in the fabric are interweaving, the cross section of the yarn will be deformed (Supplementary Figure 32b), which is generally described by the flattening coefficient (η). The flattening coefficient is the ratio of the longitudinal diameter (d_1) to the theoretical diameter (d) of the yarn cross-section. The flattening coefficient can be expressed by the following formula:

$$\eta = \frac{d_1}{d} \tag{18}$$

Woven fabrics are formed by interweaving warp and weft yarns. The warp and weft yarns are in buckling state, which the degree of flexion can be expressed by the buckling wave height (h_w) of warp and weft yarns. The distance between the peaks and troughs of the bending yarn in the fabric is called the buckling wave height. The buckling wave heights of warp and weft yarns are denoted by h_{wa} and h_{we}, respectively. In an ideal state, when the weft yarns are straight, our e-textile has the highest output performance due to the maximum degree of separation between the various frictional contact surfaces (Supplementary Figure 32c). The buckling wave height can be expressed by the following formula:

$$h_{\mathrm{wa}} = d_{\mathrm{wa}} + d_{\mathrm{we}} \tag{19}$$

where d_{wa} and d_{we} are diameter of the warp and weft yarn, respectively. However, both the warp and weft yarns in the fabric are buckling due to the existence of the flattening coefficient (Supplementary Figure 32d

and e), and their relationship can be expressed by following equation:

$$h_{\mathrm{we}} + h_{\mathrm{wa}} = \eta(d_{\mathrm{we}} + d_{\mathrm{wa}}) \qquad (20)$$

Therefore, we need to adjust parameters and tension of yarns during the weaving process so that h_{we} is close to 0 and η is close to 1.

Supplementary Note 18

In the original state, the outer and inner surfaces of the silicone rubber are charged with negative electrostatic charges, while the MPAN yarn and the inner stainless steel yarn take positive charges, respectively, due to the contact electrification. There is practically no electrical potential difference on two groups of interfaces, due to the two opposite charges coinciding at the same plane. Once the e-textile is stretched, negative charges will be induced in the outer and inner stainless steel yarn, due to electrostatic induction effect. Potential difference between two stainless steel yarn electrodes and the ground prompts electrons flowing, resulting in an electrical current. When the e-textile stops elongating, a new electrical equilibrium achieves and the electrons stop moving. When the e-textile is released and moves backward, electrons flow inversely from two stainless steel yarn electrodes to the ground to make a charge balance. When the e-textile returns to its original state, charge neutralization occurs again. To obtain a more quantitative understanding of the electricity generating process, the potential distribution of the warp-weft-connection single-electrode e-textile is simulated using COMSOL software, as demonstrated in Supplementary Figure 36b.

Supplementary Note 19

Since the weft-connection single-electrode e-textile do not collect the charge on the double-plied yarn, its output performance is slightly lower than the warp-weft-connection single-electrode e-textile. Since the friction between the silicone rubber tube and the double-plied yarn produce little charge, warp-connection single-electrode e-textile hold the lowest output performance. The double-electrode e-textile's output performance lower than the weft-connection single-electrode e-textile because a part of electric potential on the SETEY are offset the electric potential on the double-plied yarn.

Supplementary Note 20

Comparison of the compression performance of the four circuit connection patterns. As illustrated in Supplementary Figure 39a, a linear motor with a contact area of 5 × 6 cm^2 is used to trigger the e-textile, and the maximum movement distance between the external substrate and the e-textile is purposely set as 3 cm. In Supplementary Figure 39b, the electrical outputs of the four distinct patterns are shown and compared when a linear motor is used to compress the e-textile. The warp-weft-connection single-electrode e-textile's output performance slightly higher than the weft-connection single-electrode e-textile and the warp-connection single-electrode e-textile, but it shows no obvious improvement comparing with the other two patterns. This is because the charge is mainly generated by the friction between the double-plied yarns and the active object on the linear motor. Also, the double-plied yarns can generate more electrostatic charge than the SETEY due to the closer distance. The double-electrode e-textile can generate a small amount of electrical outputs, which is because the electric potential on the double-plied yarn and the electric potential on the SETEY are counteracted from each other. With the increase of the working frequency from 0.5 to 5 Hz, the output voltage rises from 31.8 to 42.1 V (Supplementary Figure 39c). For the fixed contact frequency of the 0.5 Hz, resistors are utilized as external loads to investigate the output voltage, output current, and output power. The maximum output power density reached 6.62 mW m^{-2} at a load resistance of 50 MΩ (Supplementary Figure 39d), as calculated by $P = U^2/R$, where R is the external load resistance.

Supplementary Note 21

We show that our energy harvesting textiles can charge lithium batteries under some long-term normal body movements (Supplementary Figure 41a). First, we use the device shown in Figure 4c to simulate long-term normal body movements. The Li-ion batteries (240 mAh, 3 V) are charged by energy textiles, and the battery voltage increment reaches approximately 0.36 V after continuous charging for 5 h (Supplementary Figure 41b), which indicates that energy textiles have good durability. The pre-charged lithium batteries can further power the wireless monitoring system (Supplementary Figure 41a and d), so that the body movements can be monitored in real time. As shown in Supplementary Figure 41c and Supplementary Movie 7, the energy textile is worn on the elbow of the presenter. The energy textile generates a corresponding electrical signal with the elbow bending, which is then wirelessly transmitted to the cell phone via Bluetooth. As demonstrated in Supplementary Movie 7, the different bending angles of the elbow can generate different electrical signals. Even if the bending angle is very small, there are still electrical

signals, which shows excellent sensitivity.

Supplementary Figures

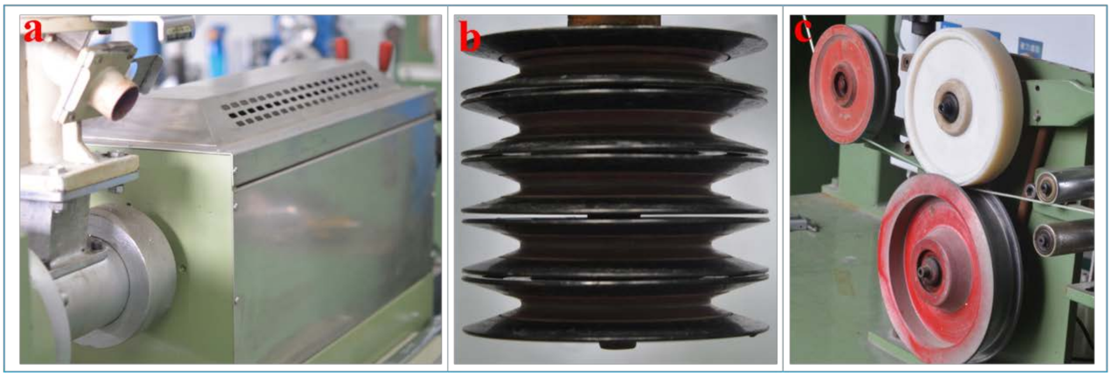

Supplementary Figure 1. Photographs of (a) the extruder, (b) the multi-groove moving pulley, and (c) the compaction roller.

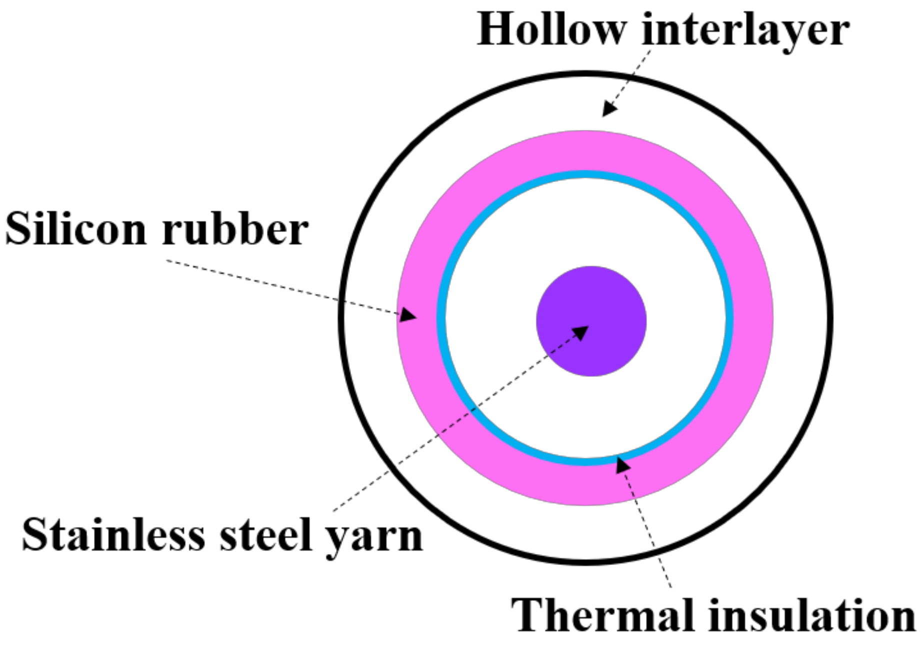

Supplementary Figure 2. The cross-section diagram of hollow equipment. The silicone rubber moves in the hollow interlayer and the stainless steel yarn moves in the center of hollow equipment.

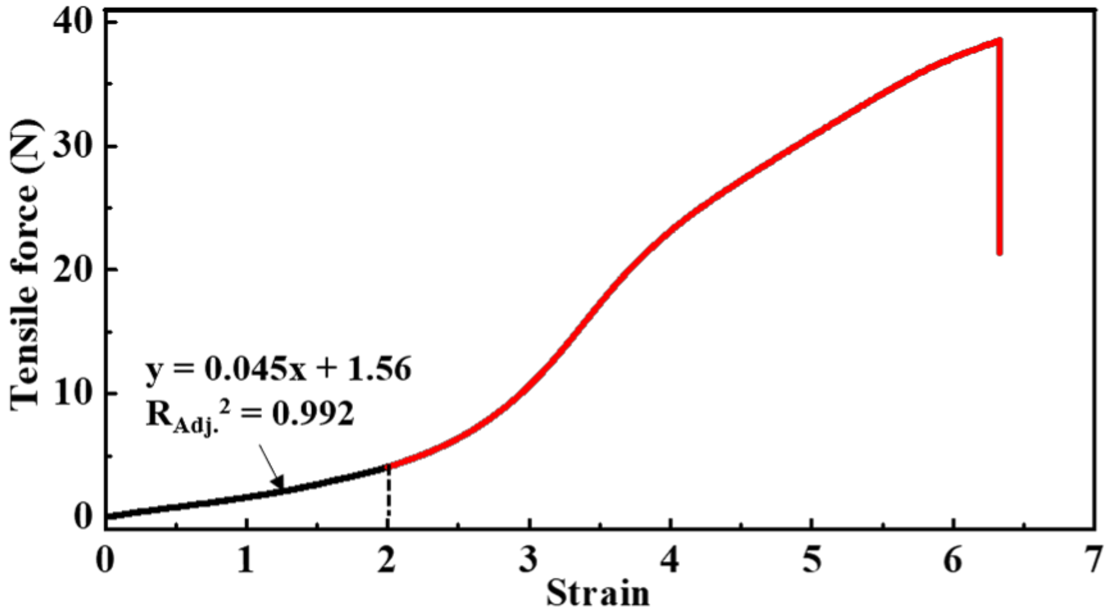

Supplementary Figure 3. Tensile force versus strain curve of silicone rubber tube. Strain can be linearly fitted to tensile force with an adjusted coefficient of determination of ≈ 0.992 under strain less than 200%. Here, the silicone rubber tube has a length of 30 cm, an inner diameter of 0.8 mm, and an outer diameter of 1.8 mm.

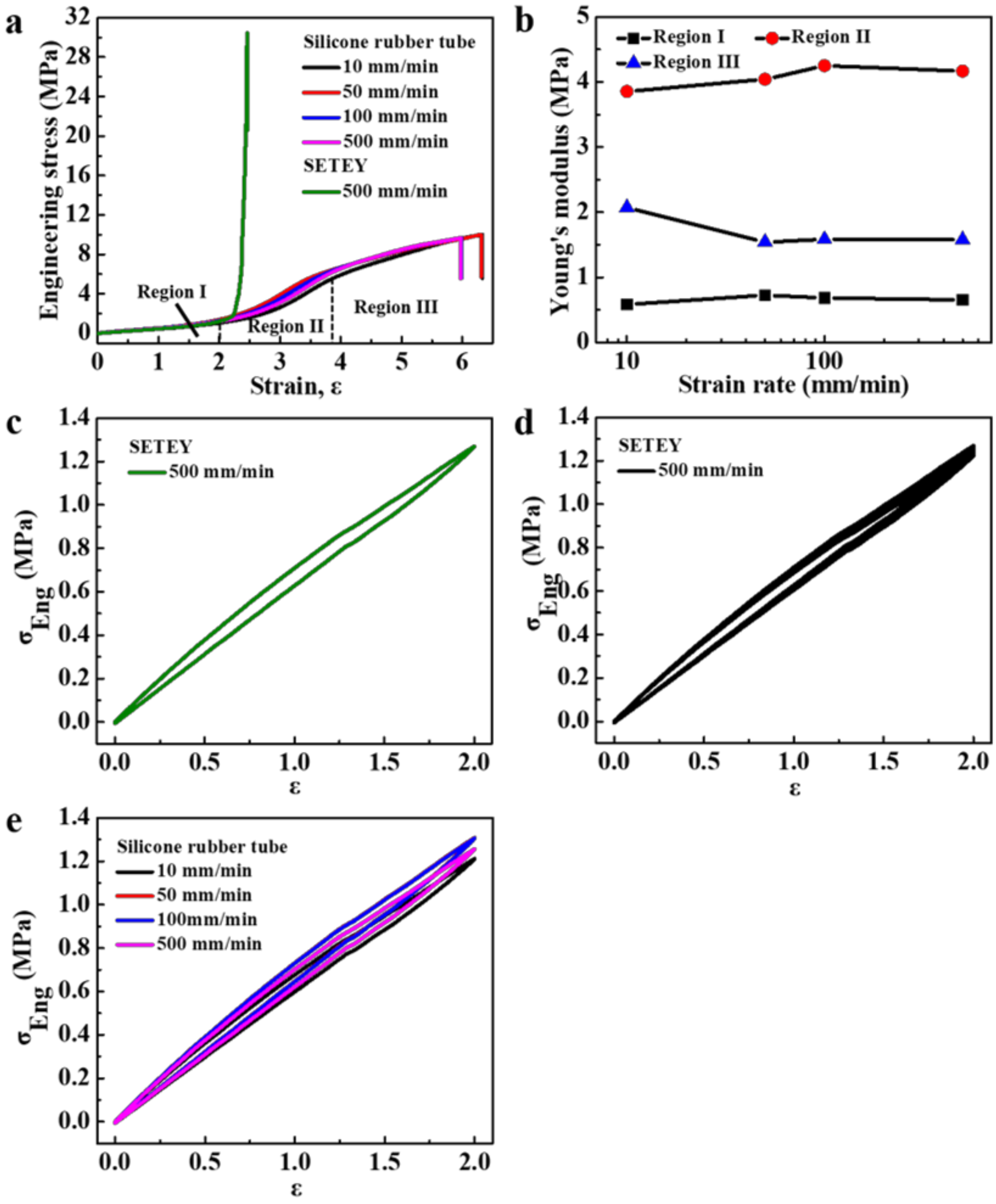

Supplementary **Figure 4.** (a) Stress-strain curves of SETEY measured at different strain rates. (b) Young's modulus of SETEY measured at different strain rates in three regions: small deformation (Region I, squares), intermediate deformation (Region II, circles), and large deformation (Region III, triangles). (c) Single cyclic tensile test of SETEY at a strain of 200% under the deformation rate of 500 mm/min. (d) Cyclic tensile tests (10 cycles) of SETEY at a strain of 200% under the deformation rate of 500 mm/min. The SETEY reveals little hysteresis in the cyclic tensile tests, which indicates good elastic deformation of SETEY. (e) Single cyclic tensile test of silicone rubber tube at a strain of 200% under different deformation rate.

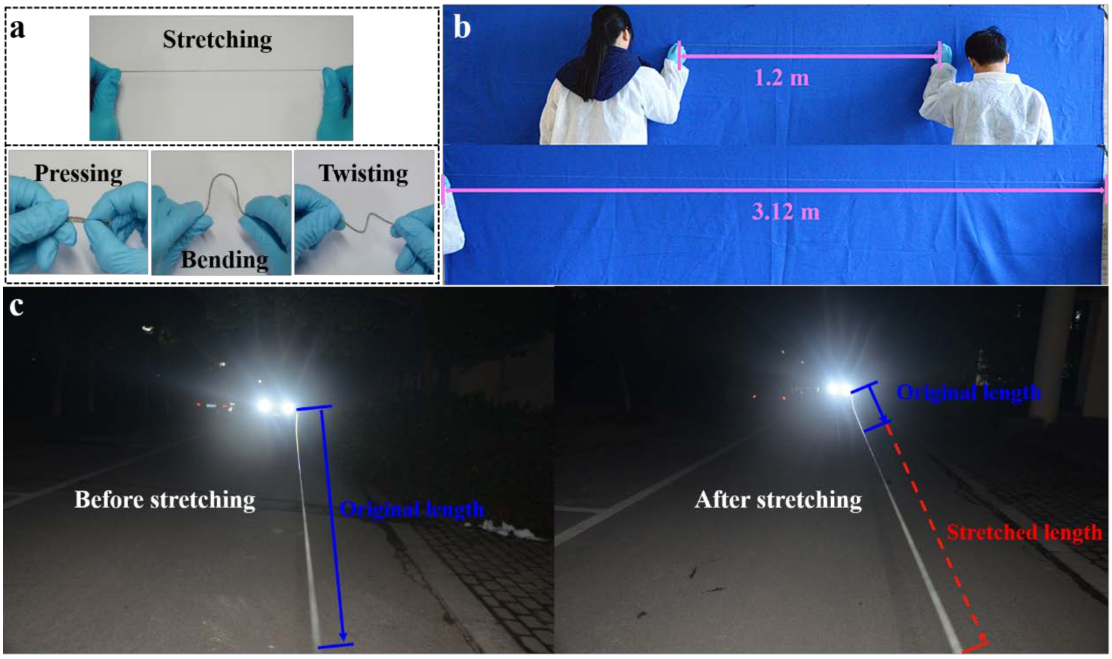

Supplementary Figure 5. (a) Photographs of SETEY with demonstrations of being different mechanical forces including stretching, pressing, bending, and twisting. (b) and (c) Photographs of short and long SETEYs before and after stretching, respectively.

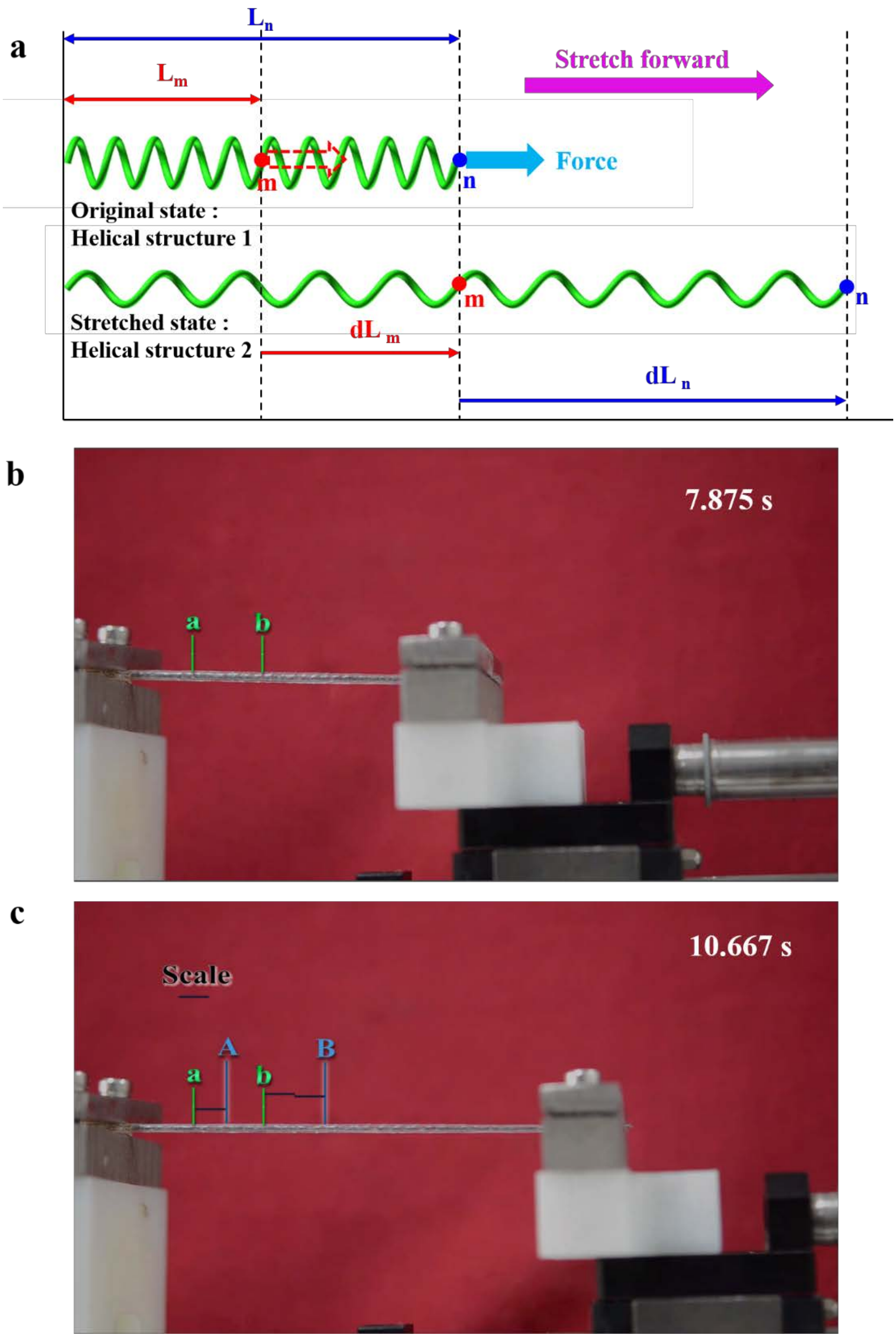

Supplementary Figure 6. (a) Schematic diagram of the helical structure from the original state to the stretched state. It is worth noting that the helical structure 1 and the helical structure 2 are different states of the same object. (b) An optical photograph of a triboelectric yarn at 8.875 s, where the marks a and b are the two helices at 1/4 and 1/2 of the triboelectric yarn, respectively. (c) An optical photograph of a triboelectric yarn at 10.667 s, wherein the marks A and B are positions where the marks a and b are stretched, respectively, and the black line is used as a scale to indicate the distance before and after stretching.

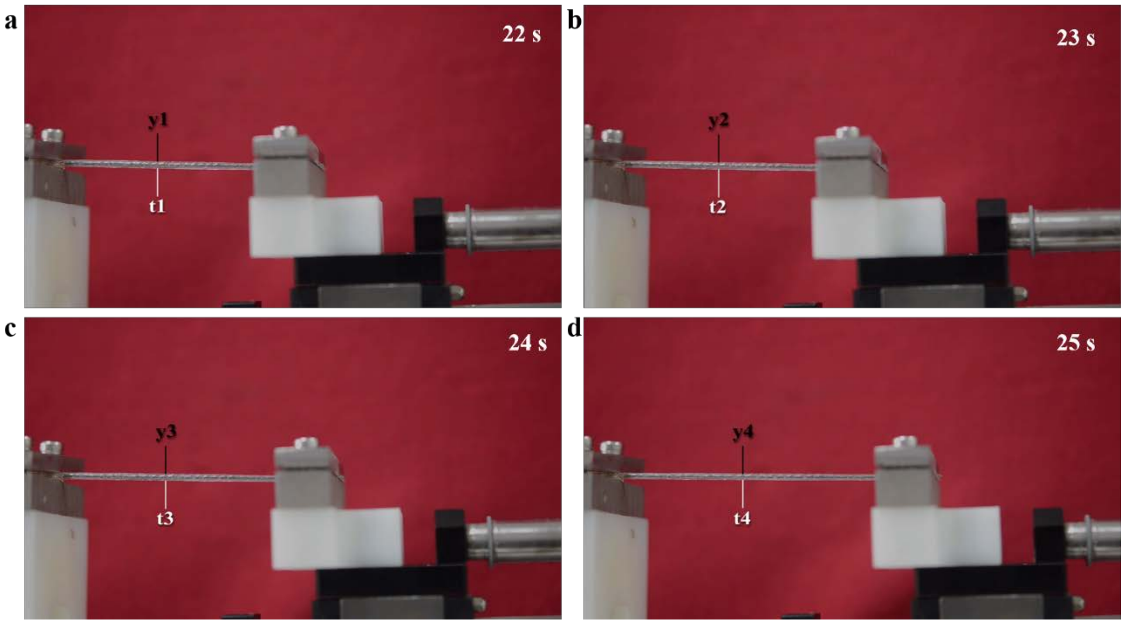

Supplementary Figure 7. During stretching, the triboelectric yarns were photographed at (a) 22 s, (b) 23 s, (c) 24 s, and (d) 25 s, respectively, where stainless steel yarn and silicone rubber tube were marked with y and t, respectively.

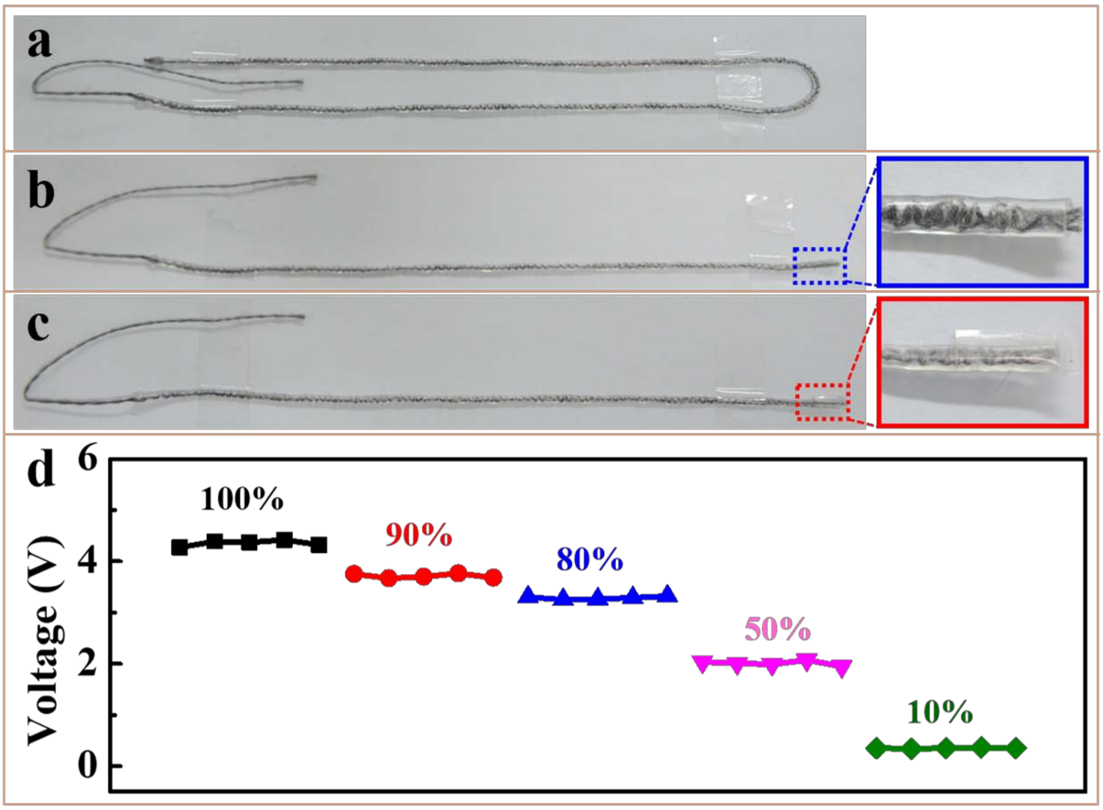

Supplementary Figure 8. Photographs of a SETEY test specimen (a) before cutting, (b) before sealing, (c) after sealing. (d) The voltages of SETEY's remainders, remaining ratios are 100%, 90%, 80%, 50%, 10%, respectively.

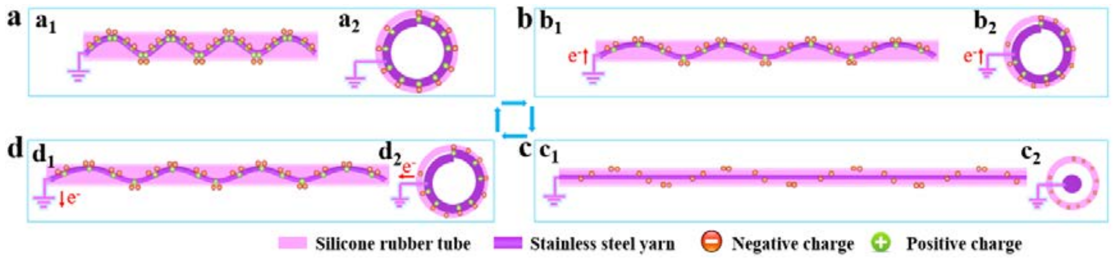

Supplementary Figure 9. Schematic diagram of the working mechanism of SETEY's (a_1-d_1) front view and (a_2-d_2) side view, respectively.

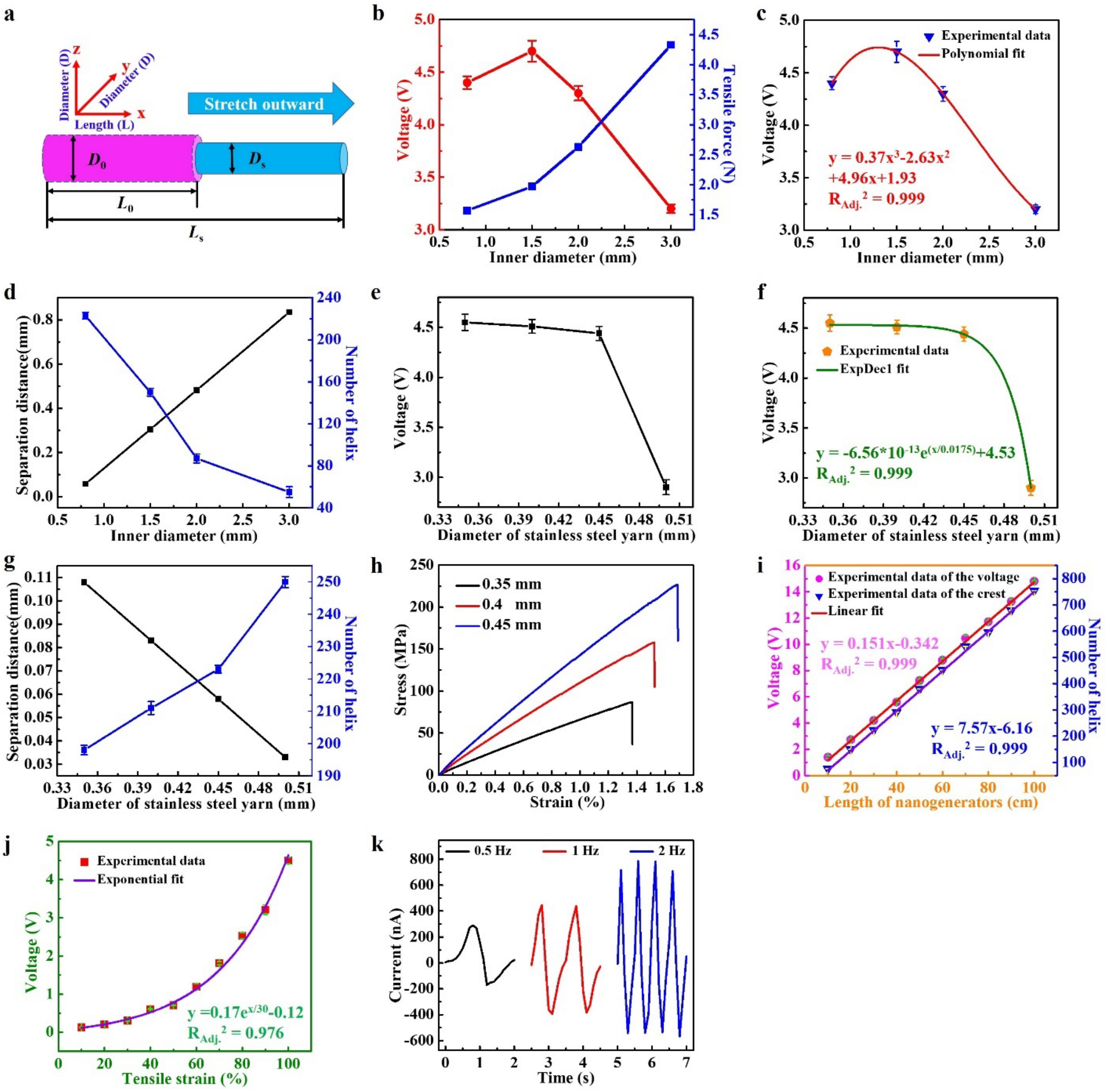

Supplementary Figure 10. (a) Schematic depiction of the silicone rubber tube being stretched outward. (b) Voltage and tensile force versus inner diameter curves of SETEY measured under 100% tensile strain. (c) The fitting relationship between the inner diameter of the silicone rubber tube and the output voltage of the SETEY when the diameter of the stainless steel yarn is 0.45 mm, the length of the SETEY is 30 cm and the tensile strain is 100%. (d) The separation distance and the number of helix vary with the inner diameter of the silicone rubber tube when the diameter of the stainless steel yarn is 0.45 mm. (e) Voltage versus the diameter of the stainless steel yarn curves measured under 100% tensile strain. (f) The fitting relationship between the diameter of the stainless steel yarn and the output voltage of the SETEY when the inner diameter of the silicone rubber tube is 0.8 mm, the length of the SETEY is 30 cm and the tensile strain is

100%. (g) The separation distance and the number of helix vary with the diameter of the stainless steel yarn when the inner diameter of the silicone rubber tube is 0.8 mm. (h) Tensile stress versus strain curve of the stainless steel yarns of different diameters (diameter: 0.35mm, 0.4mm, 0.45mm). (i) The summarized changes of the voltage and the number of helix under different length of SETEY. (j) Relationship between the output voltage and tensile strain at fixed parameters including a 0.5 Hz stretching frequency, a length of 30 cm, and an external load of 20 MΩ. (k) The measured output current under different arm bending frequency. The error bars correspond to standard deviation caused by the measurement noise.

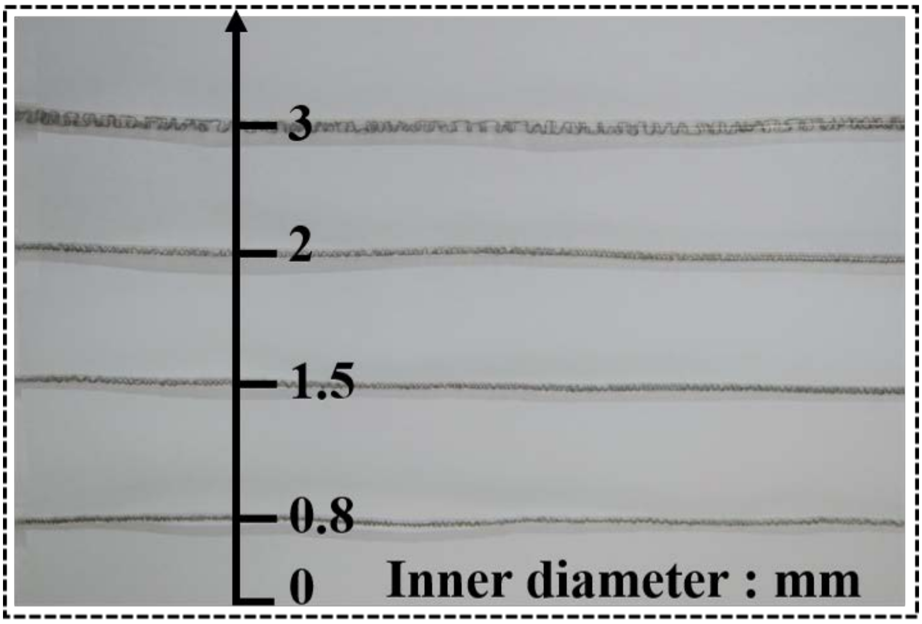

Supplementary Figure 11. Photograph of SETEYs with different inner diameters. SETEYs with different inner diameters (0.8, 1.5, 2, and 3 mm) are compared to their practicality under 100% tensile strain. The inner diameter-dependent the output voltage and tensile force are plotted.

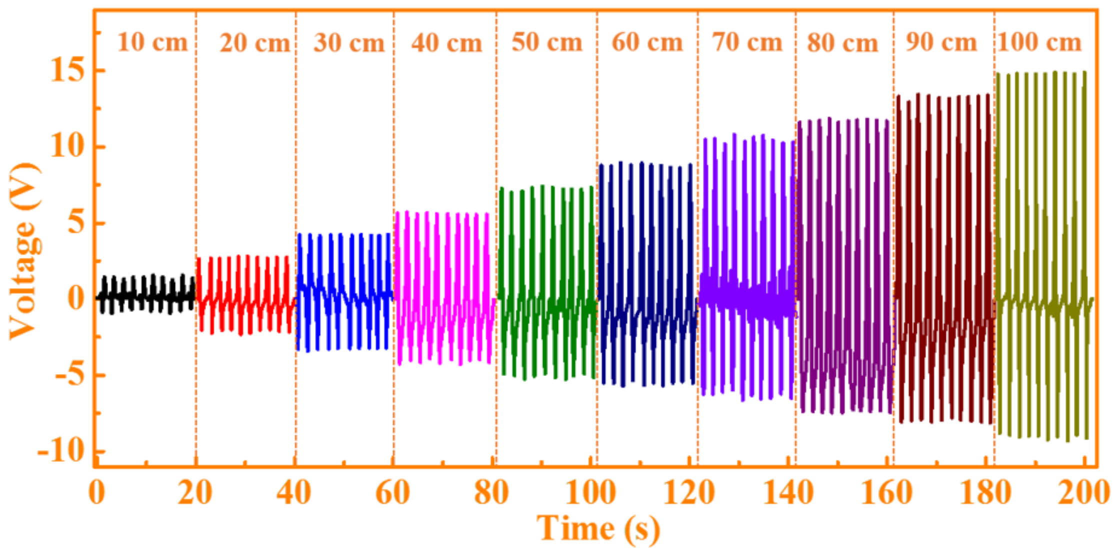

Supplementary Figure 12. The measured output voltage under different length of SETEY, at a fixed tensile strain of 100%.

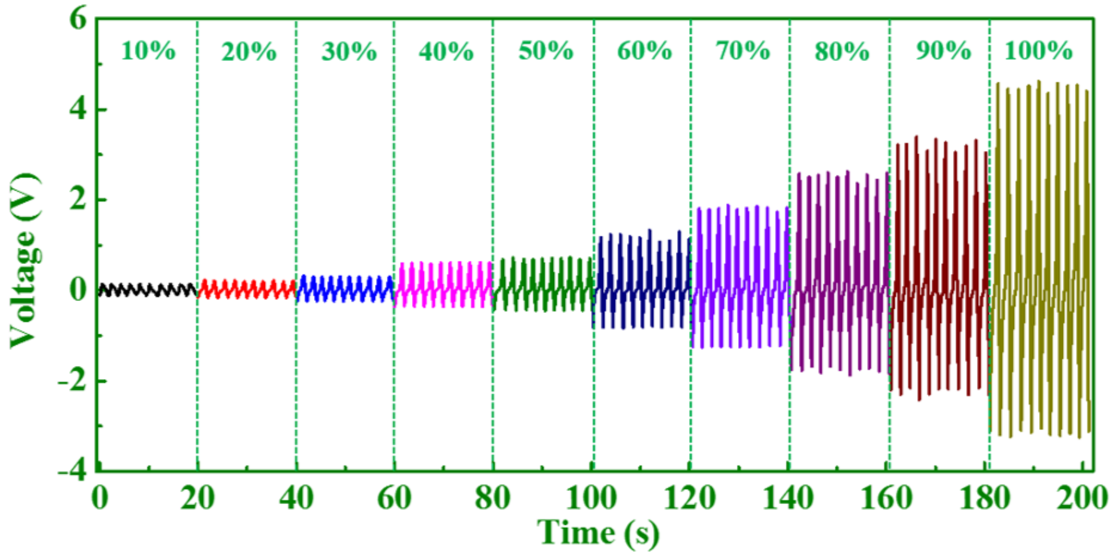

Supplementary Figure 13. The measured output voltage of a 30-cm-length SETEY measured under different tensile strain.

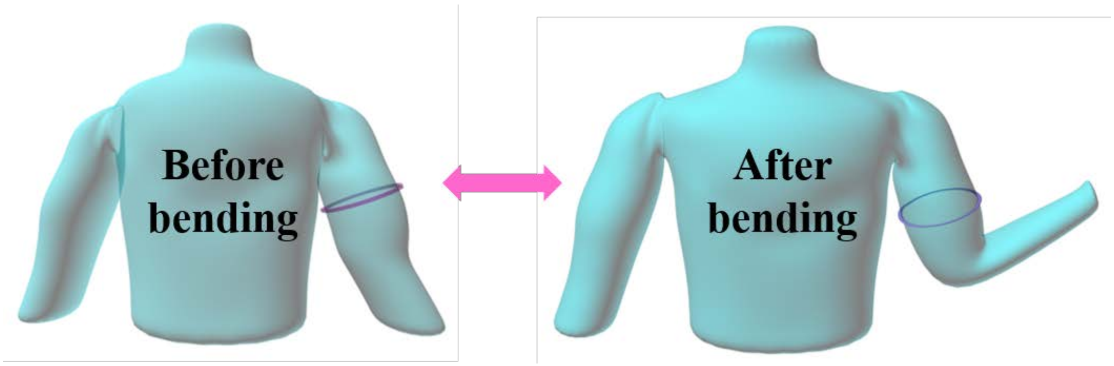

Supplementary Figure 14. Schematic diagram of muscle changes during arm bending.

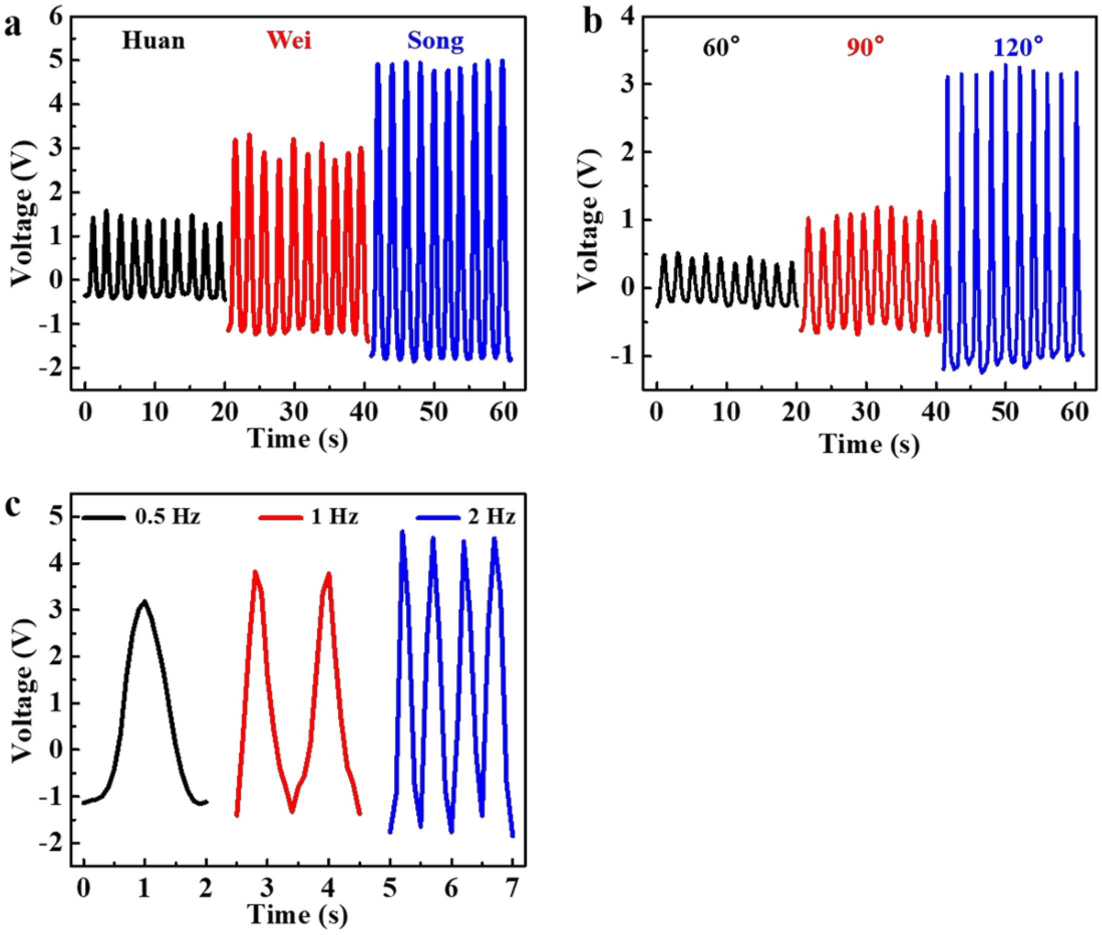

Supplementary Figure 15. The applications of SETEY as self-powered active sensors for monitoring of muscle changes during arm bending. (a) The output signals are recorded when three persons (Huan, Wei, Song) with wearing the same equipment bend their arms to 120°, respectively. The arm muscles of the three persons are different, then the tensile strain of the SETEYs are diverse when their arm is bent, so a variety of output signals would appear. (b)The output voltage of the SETEY obviously increase with increasing bending angle of Wei's arm at frequency of 0.5 Hz. (c) The voltage of the SETEY slightly increase when the bending frequency of arm vary from 0.5 to 2 Hz.

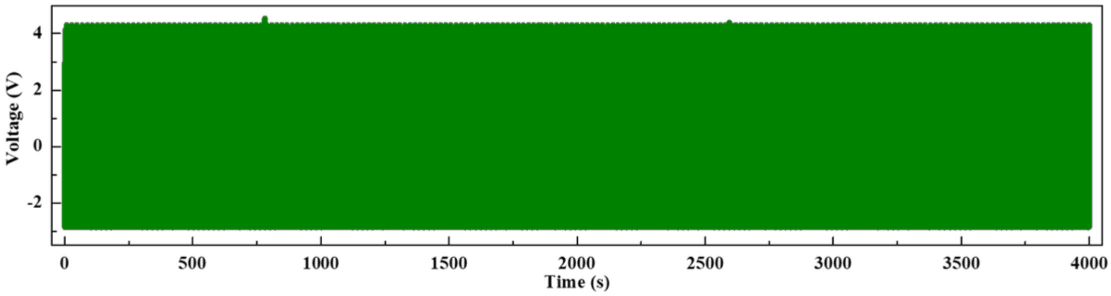

Supplementary Figure 16. Stability and reliability measurement of SETEY, where the voltage was recorded for 2000 cycles at a strain of 100% and a frequency of 0.5 Hz.

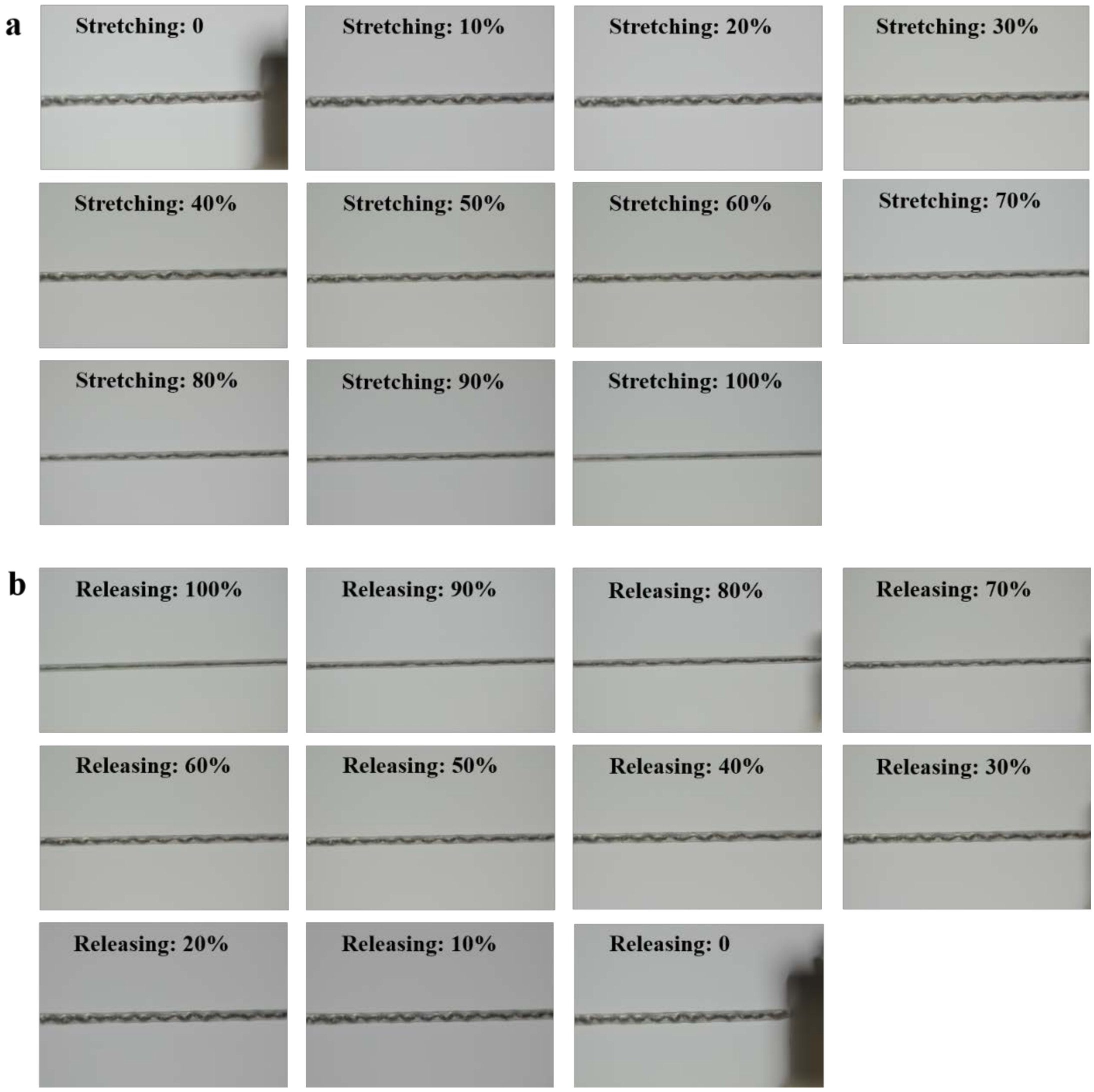

Supplementary Figure 17. The longitudinal morphologies of SETEY under different strains during (a) stretching and (b) releasing. The built-in helix structure always exists as the tensile strain increases before the SETEY is fully stretched (i.e., the working strain is 100%). During the stretching and releasing process, the SETEY under the same strain has almost the same longitudinal morphology, which indicates that the morphological change of SETEY has good repeatability.

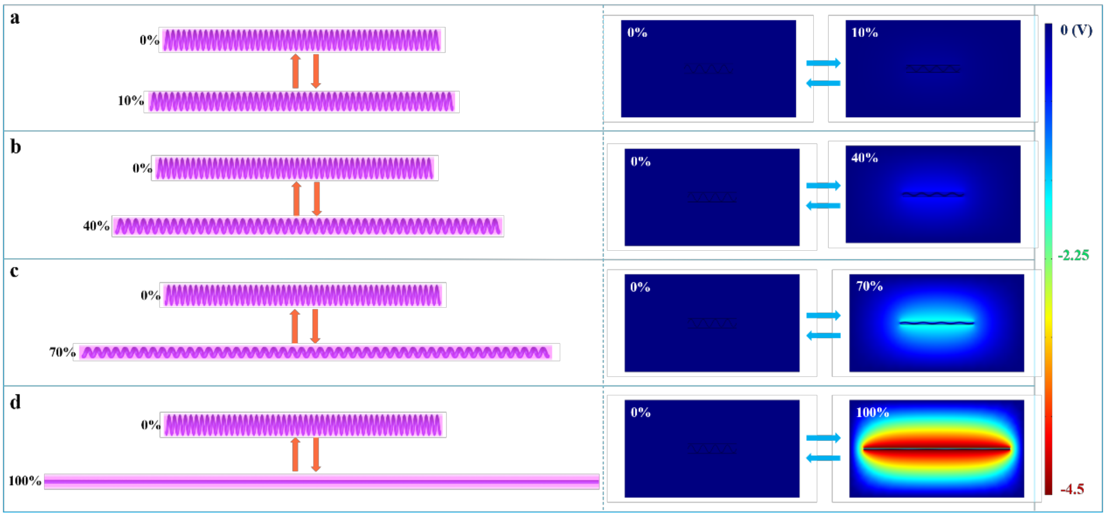

Supplementary Figure 18. Numerical calculations of potential distribution when the SETEY are stretched to strains of (a) 10%, (b) 40%, (c) 70%, (d) 100% from their initial state, respectively. The separation distance between the silicone rubber tube and the helical stainless steel yarn during the process of separation increases exponentially with increasing tensile strain, thereby potential distribution increases exponentially with increasing strain.

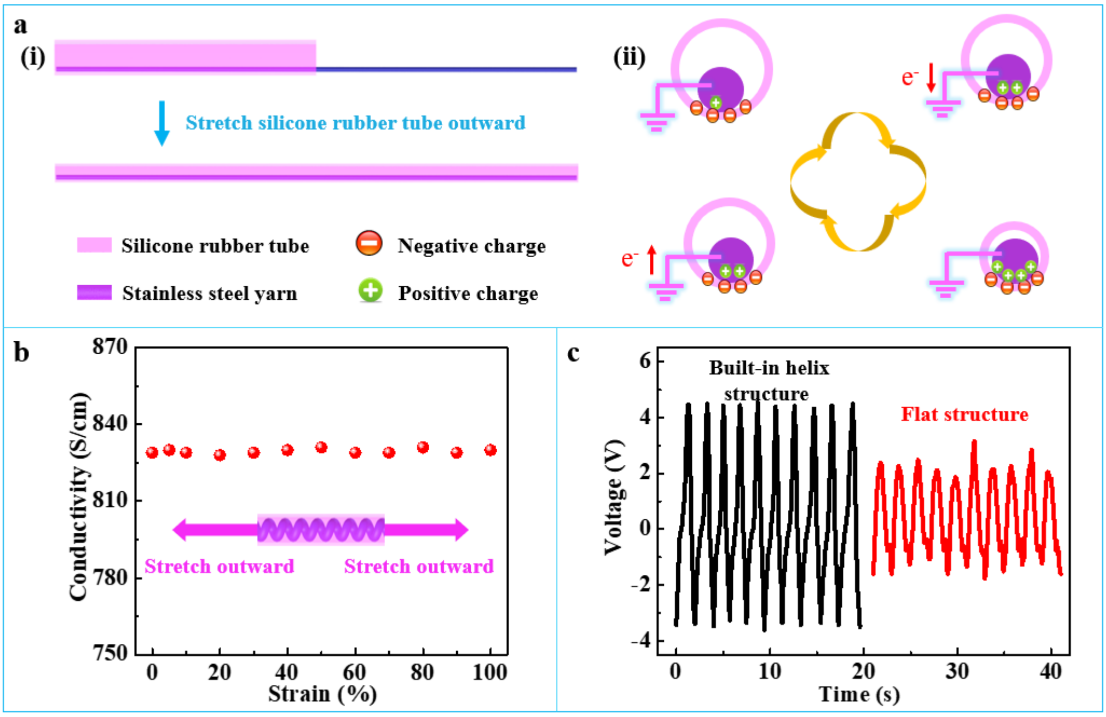

Supplementary Figure 19. (a) The structure and mechanism of a core-shell yarn-like TENG with a flat core yarn. (i) Schematic description of the core-shell yarn-like TENG with the silicone rubber tube stretched outward. (ii) Schematic diagram of the working principle of the core-shell yarn-like TENG under the stretching state. (b) The conductivity variation of the core electrode of the SETEY under stretching test. (c)The output voltage of built-in helix structure and flat structure at a fixed tensile strain of 100%.

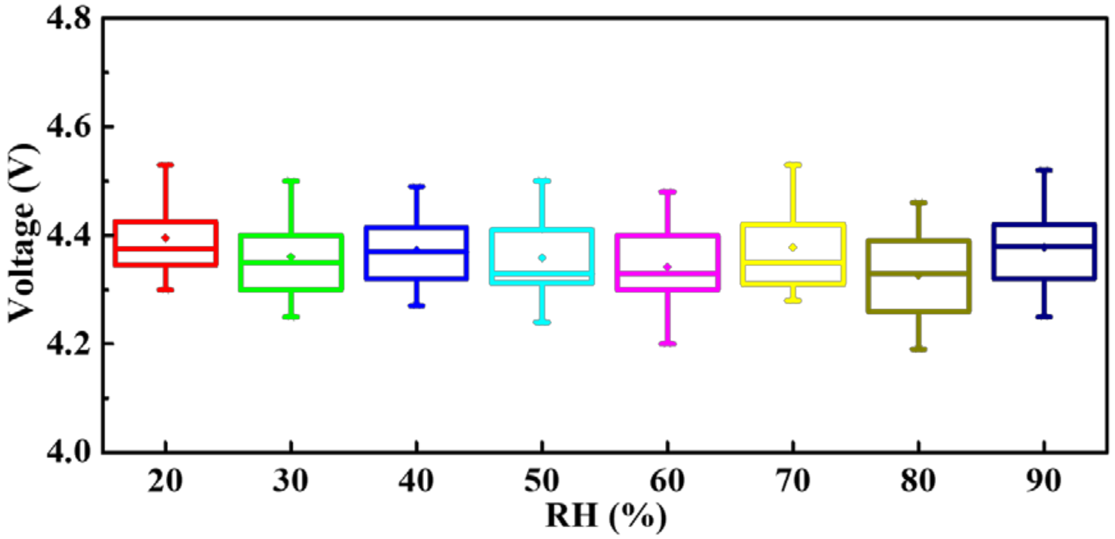

Supplementary Figure 20. The output voltage of the SETEY is measured under different relative humidity (RH) with an external load resistance of 20 MΩ. The humidity of environment does not affect the charge transfer during the contact and separation of two materials inside the silicone rubber tube. This is owing to the hydrophobicity of the silicone rubber tube surface which makes it difficult to form a close water layer on silicone rubber tube surface even in a high-humidity environment. This unique property promises the SETEY serviceability in different environments.

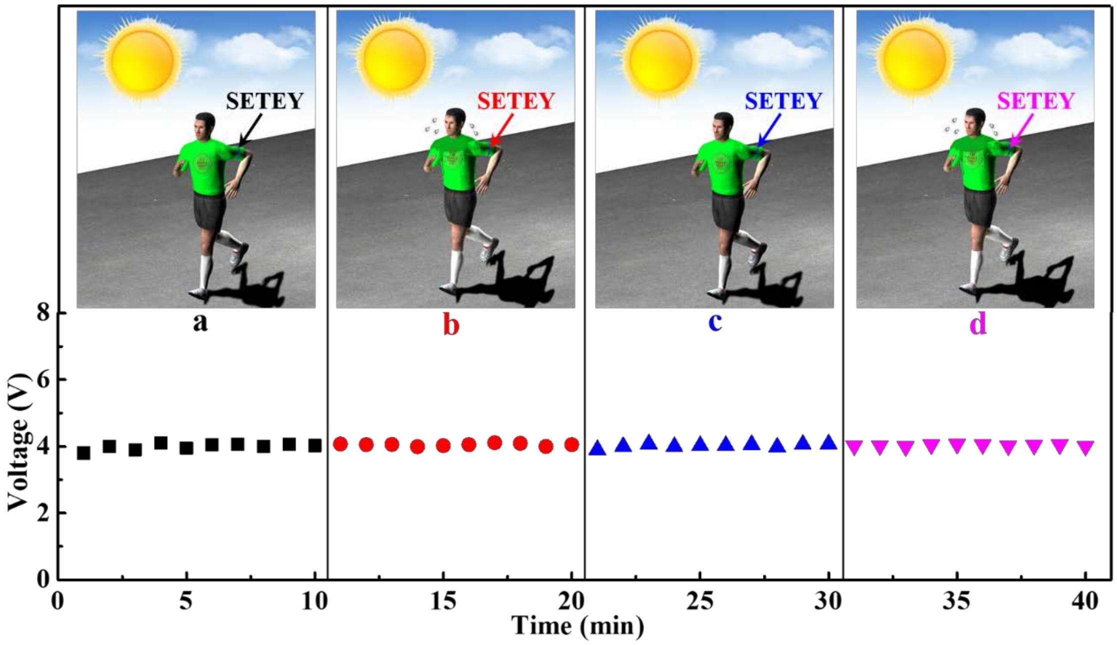

Supplementary Figure 21. The output signals of the SETEY during exercise when it is worn on the athlete's arm. The output voltages of (a) SETEY when the athlete starts running, (b) SETEY when the athlete sweats, (c) SETEY when the athlete runs again after the break, and (d) SETEY when the athlete sweats again. It can be seen that the output signals of the SETEY is not affected by sweat.

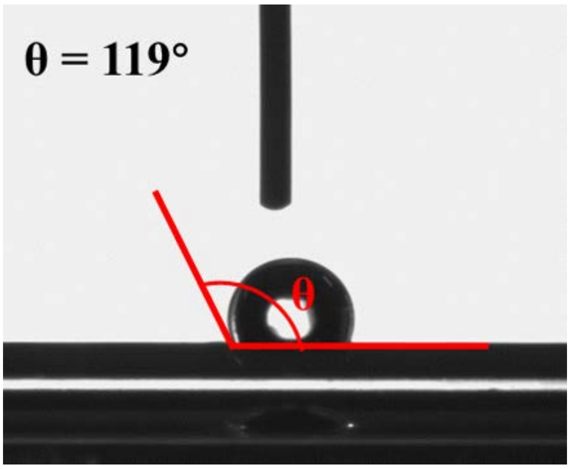

Supplementary Figure 22. The contact angle analysis of the silicone rubber tube.

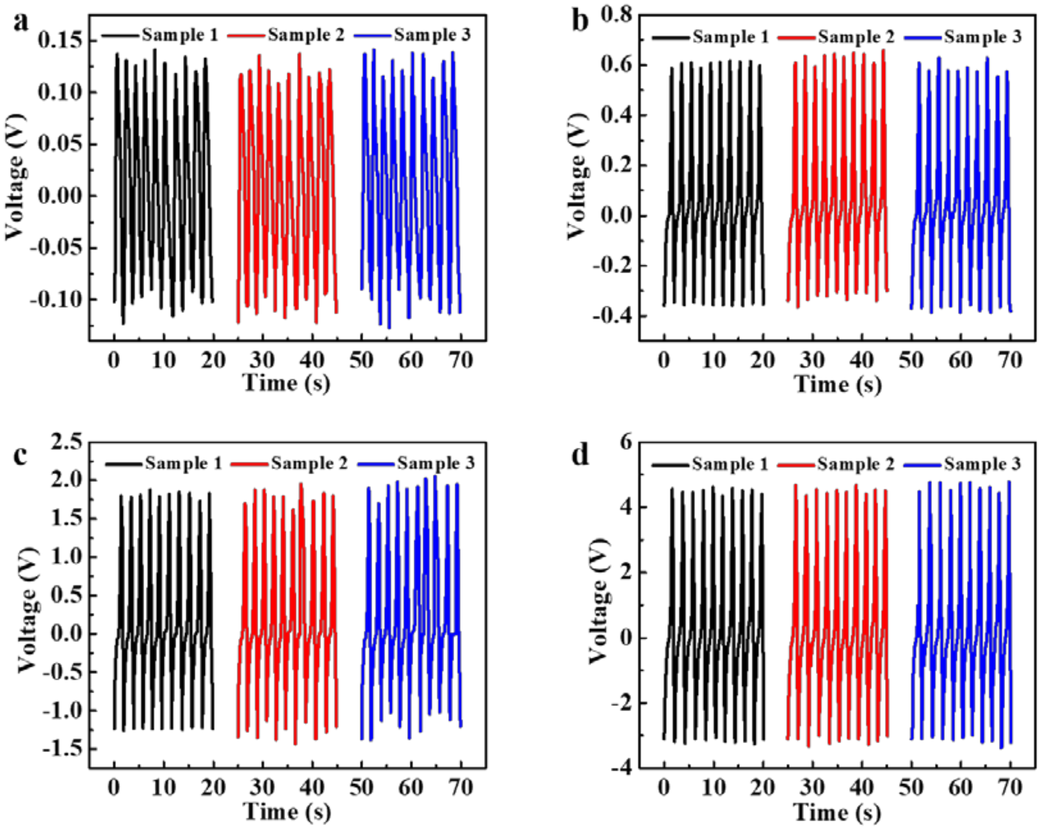

Supplementary Figure 23. The output voltage comparison of three samples 1, 2 and 3. (a) Output voltages under stretching strain of 10%; (b) Output voltages under stretching strain of 40%; (c) Output voltages under stretching strain of 70%; (d) Output voltages under stretching strain of 100%.

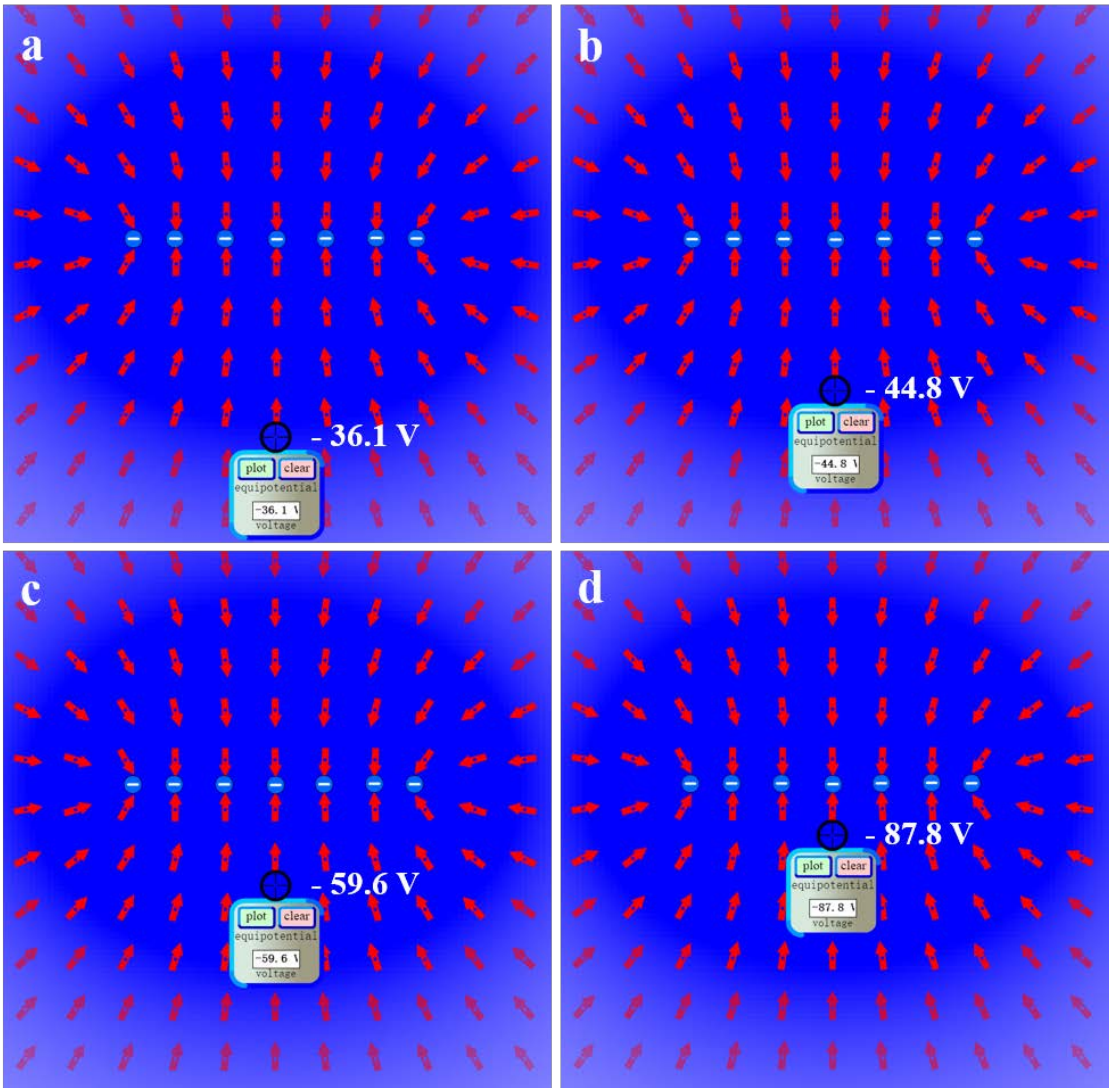

Supplementary Figure 24. Simulation results of the electric field distribution of the point charge group by using Charges and Fields software. From (a) to (d): the potential increases as the distance to the point charge group gradually decreases.

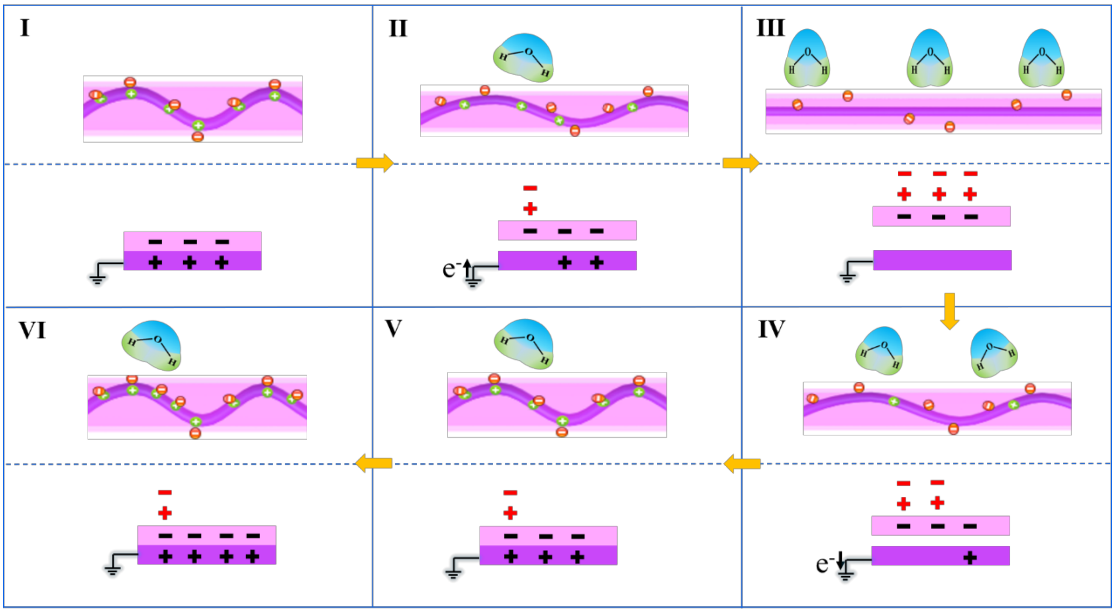

Supplementary Figure 25. The schematic illustration of working mechanism of SETEY under water.

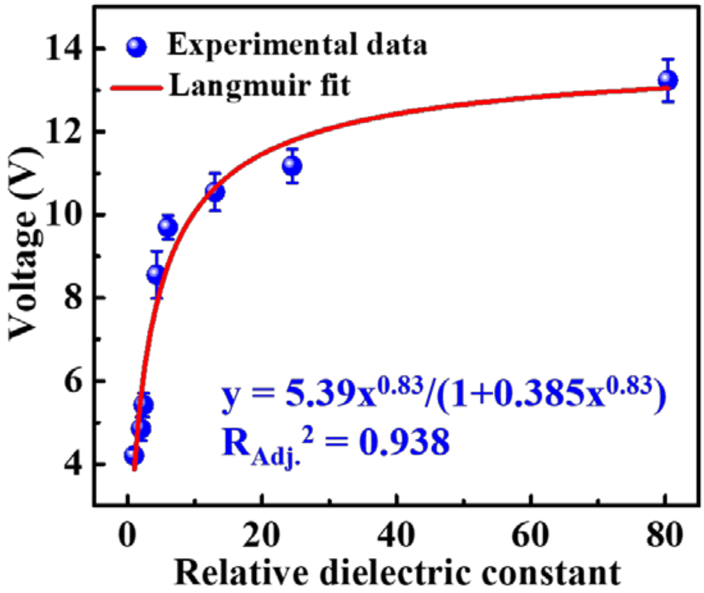

Supplementary Figure 26. Relationship between the relative dielectric constant of the liquid and the output voltages of the triboelectric yarn. The error bars correspond to standard deviation caused by the measurement noise.

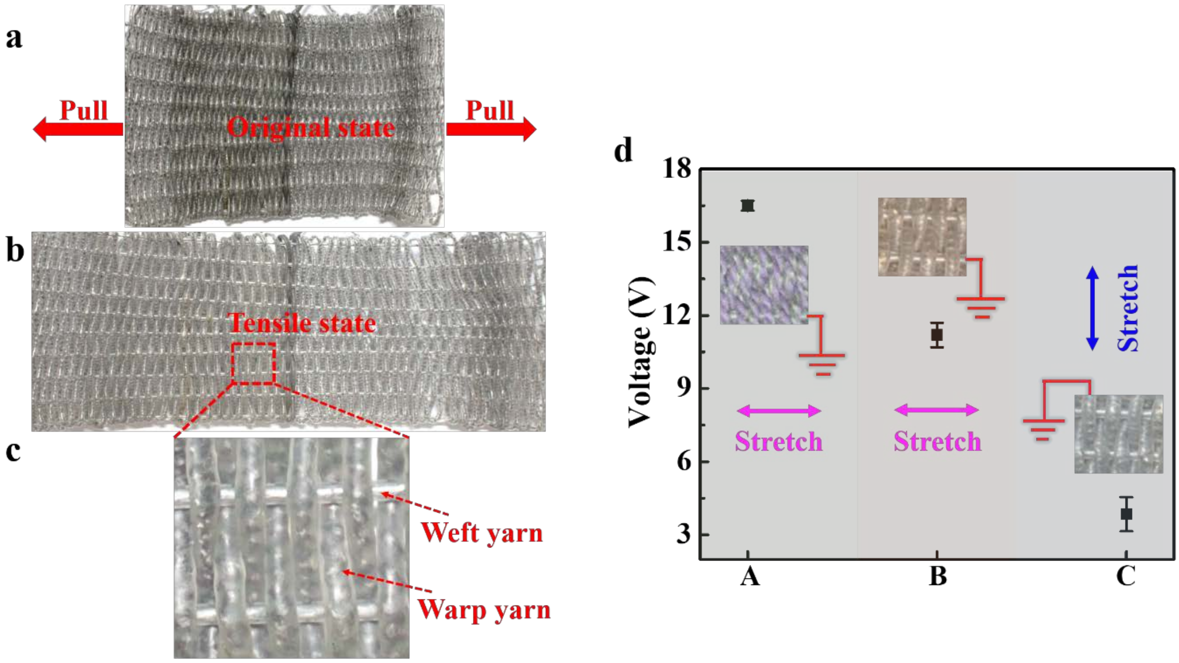

Supplementary Figure 27. Optical photographs of the TENG fabric woven from two SETEYs in its (a) original state, (b) tensile state and (c) enlarged view, respectively. (d) A is the output voltage of the weft yarn of the TENG fabric woven from a SETEY and a MPAN yarn. B is the output voltage of the weft yarn of the TENG fabric woven from two SETEYs. C is the output voltage of the warp yarn of the TENG fabric woven from two SETEYs. The error bars correspond to standard deviation caused by the measurement noise.

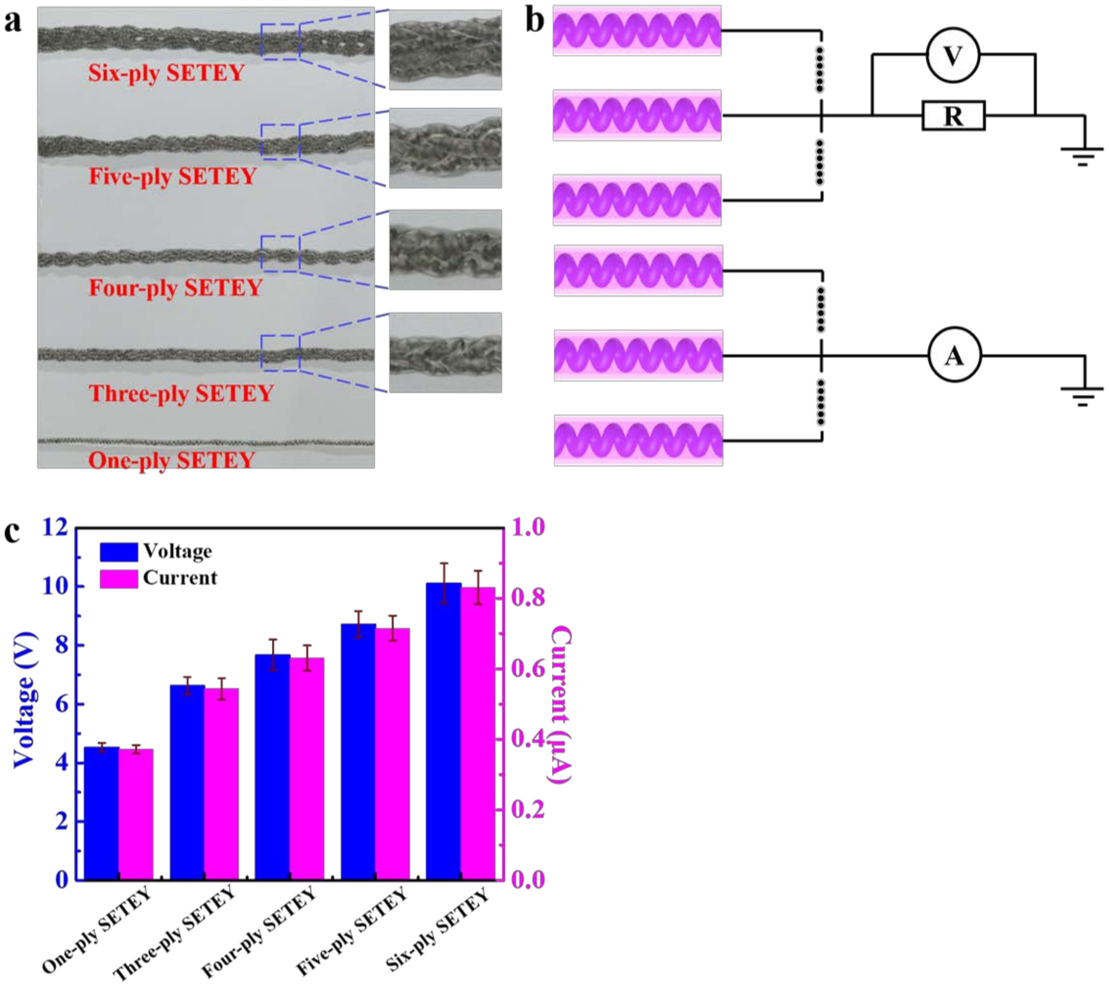

Supplementary Figure 28. (a) The photographs of n-ply devices of SETEY (n = 1, 3, 4, 5 or 6). (b) The circuit diagrams of n-ply device of SETEY for voltage and current testing. (c) The measured values of voltage and current of n-ply devices. The error bars correspond to standard deviation caused by the measurement noise.

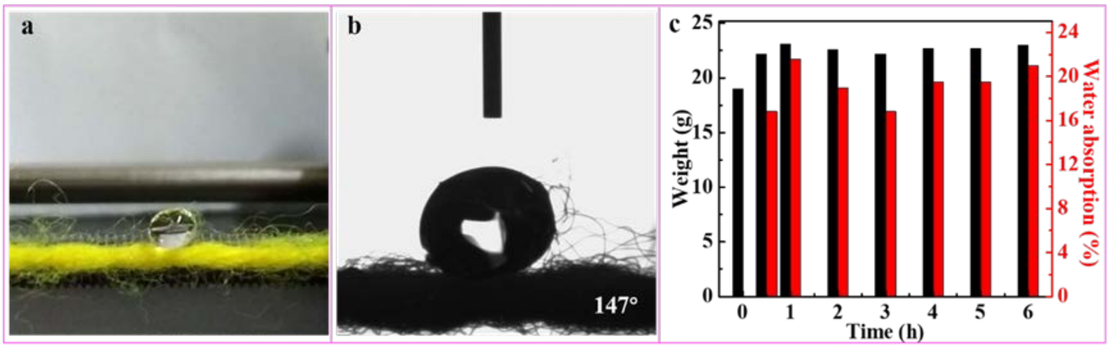

Supplementary Figure 29. (a) Optical images showing a macroscopic water droplet on the MPAN yarn. (b) The contact angle analysis of the MPAN yarn. (c) The ordinate on the left is weight of the TENG yarn before and after absorbing water, and the ordinate on the right is analysis of the water absorption of the TENG yarn. So e-textile has the potential to run underwater.

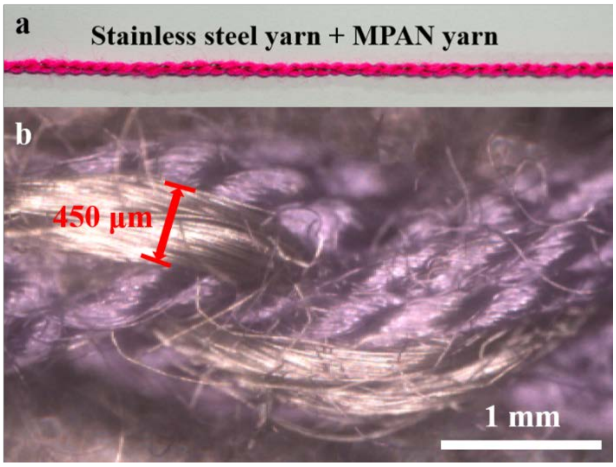

Supplementary Figure 30. (a) Digital photo and (b) optical microscope photograph of the double-plied yarn.

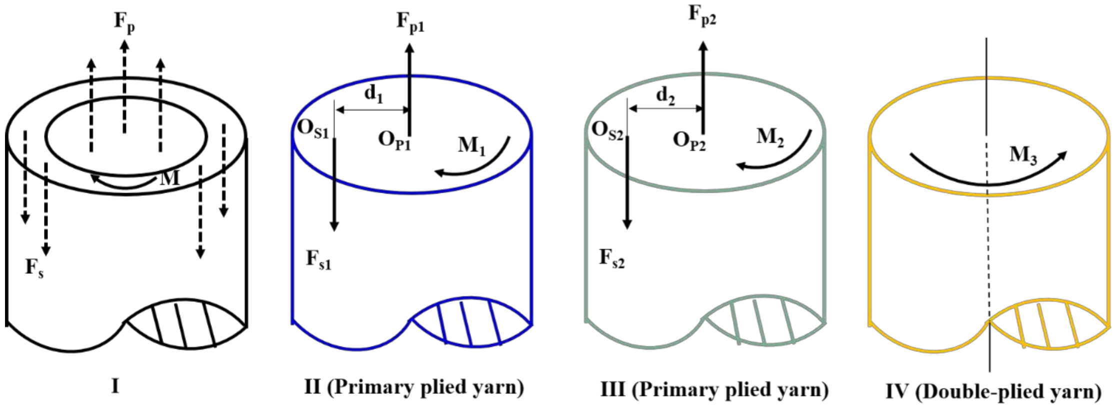

Supplementary Figure 31. Structure design of the double-plied yarn, including (I) force diagram of the ordinary plied yarn, (II, III) force diagram of two primary plied yarn, and (IV) force diagram of the double-plied yarn.

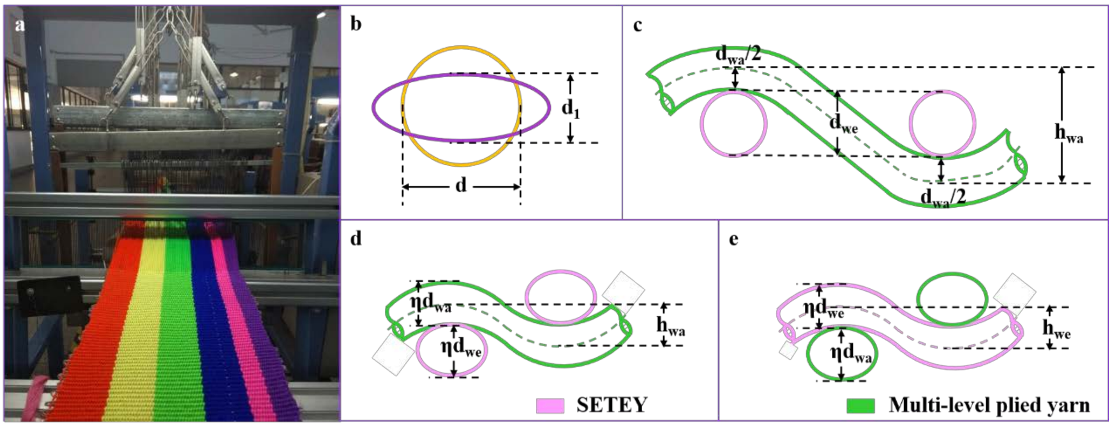

Supplementary Figure 32. (a) Digital photo of the weaving process of the e-textile. (b) Diagram of yarn squashed state. (c) The schematic diagram of warp yarn buckling in the ideal state. (d, e) The schematic diagrams of warp and weft yarn buckling under general condition, respectively.

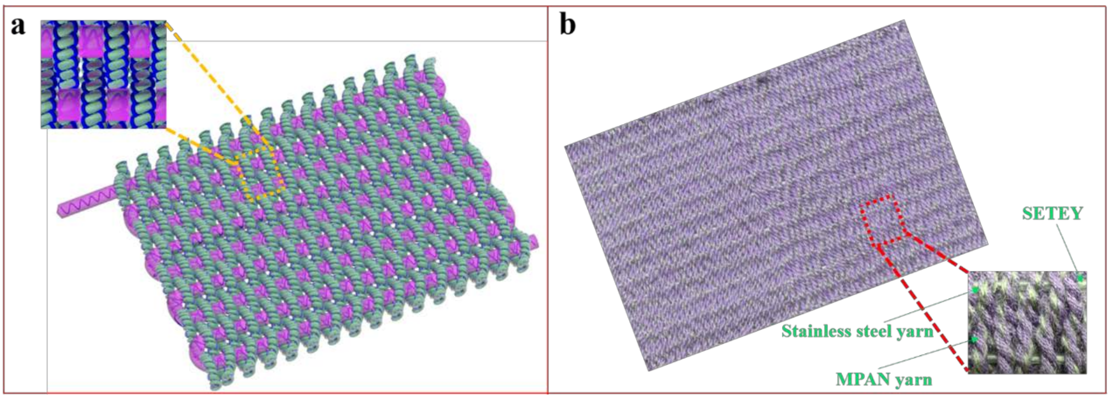

Supplementary Figure 33. (a) Schematic illustration and (b) digital photograph of the e-textile. The top left and bottom right are the partial enlarged top views of schematic illustration and digital photograph, respectively.

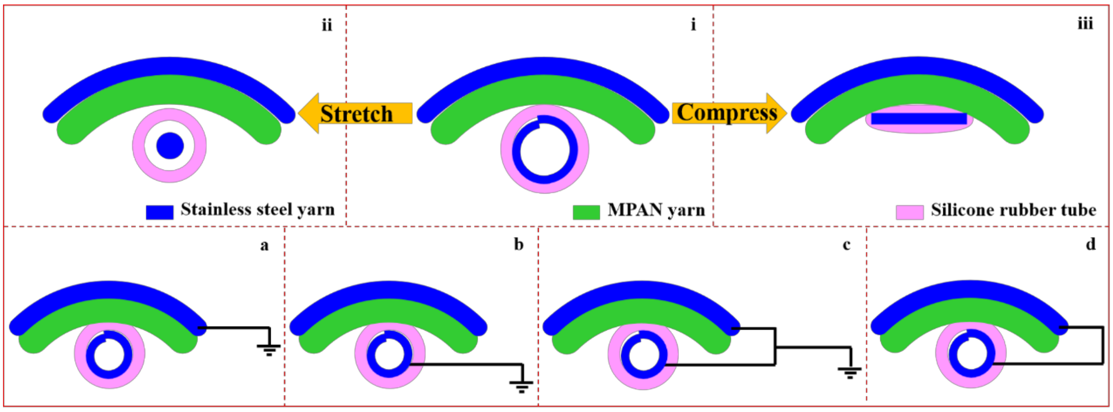

Supplementary Figure 34. Cross-section diagrams of e-textiles, including (i) initial state of the e-textile, (ii) state of the e-textile after being stretched, (iii) state of the e-textile after being compressed, (a) warp-connection single-electrode pattern, (b) weft-connection single-electrode pattern, (c) warp-weft-connection single-electrode pattern, and (d) double-electrode pattern.

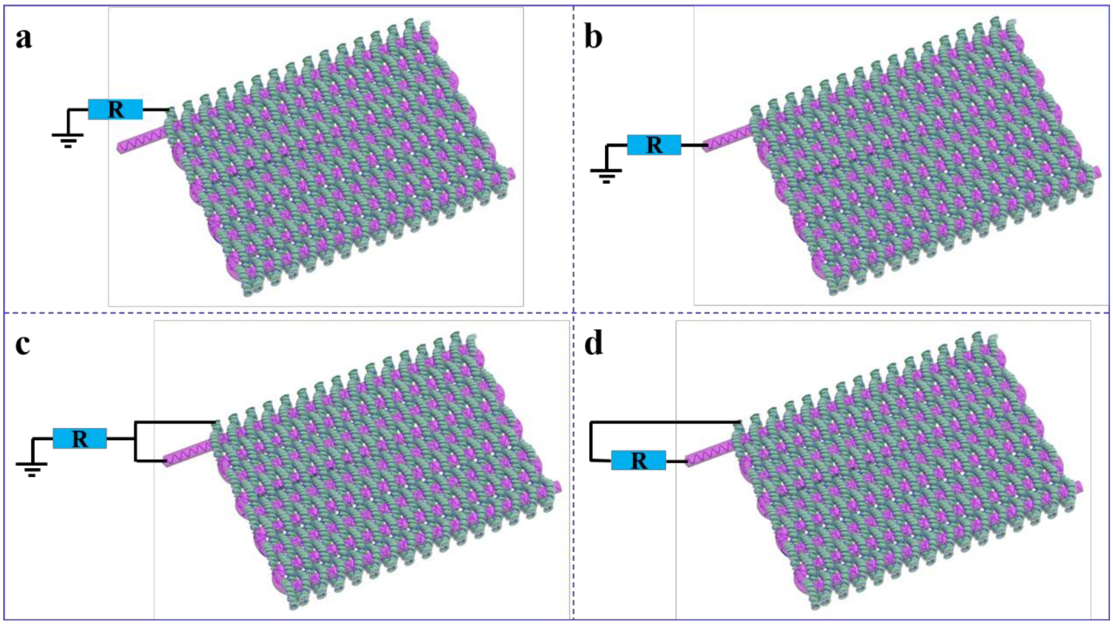

Supplementary Figure 35. Schematic illustrations of four types of circuit connection patterns of the e-textiles, including (a) warp-connection single-electrode pattern, (b) weft-connection single-electrode pattern, (c) warp-weft-connection single-electrode pattern, and (d) double-electrode pattern.

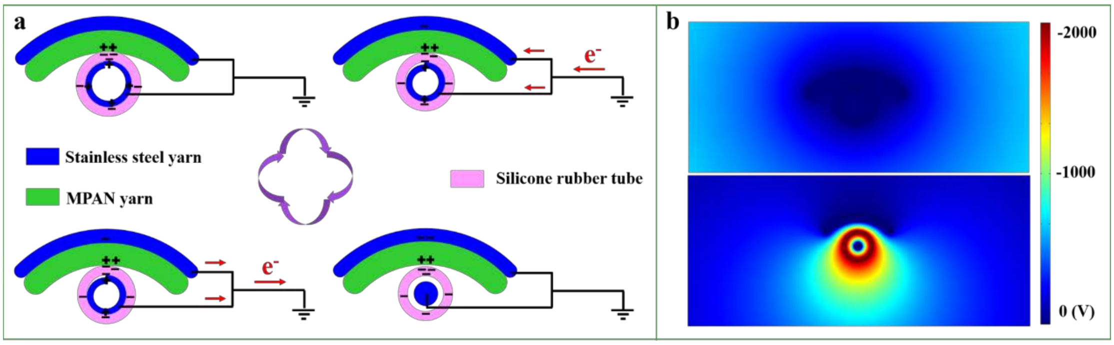

Supplementary Figure 36. The working mechanism of the e-textile in normal circumstances. (a) Schematic diagrams of the working principles of the warp-weft-connection single-electrode e-textile under contact-separation motion. (b) Finite element simulation of the potential distribution in warp-weft-connection single-electrode e-textile.

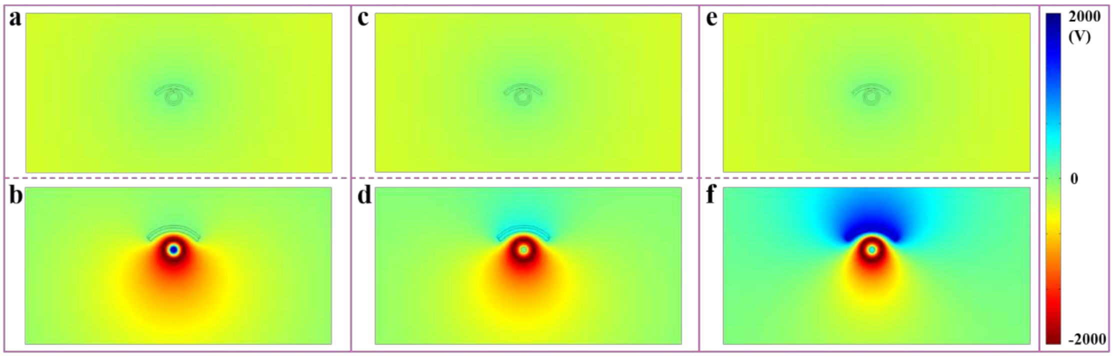

Supplementary Figure 37. Simulation results of the potential distribution of (a, b) warp-connection single-electrode e-textile, (c, d) weft-connection single-electrode e-textile, (e, f) double-electrode e-textile by using COMSOL software. (a, c, and e) are initial states of the e-textile, and (b, d, and f) are potential distributions of the e-textile after stretching.

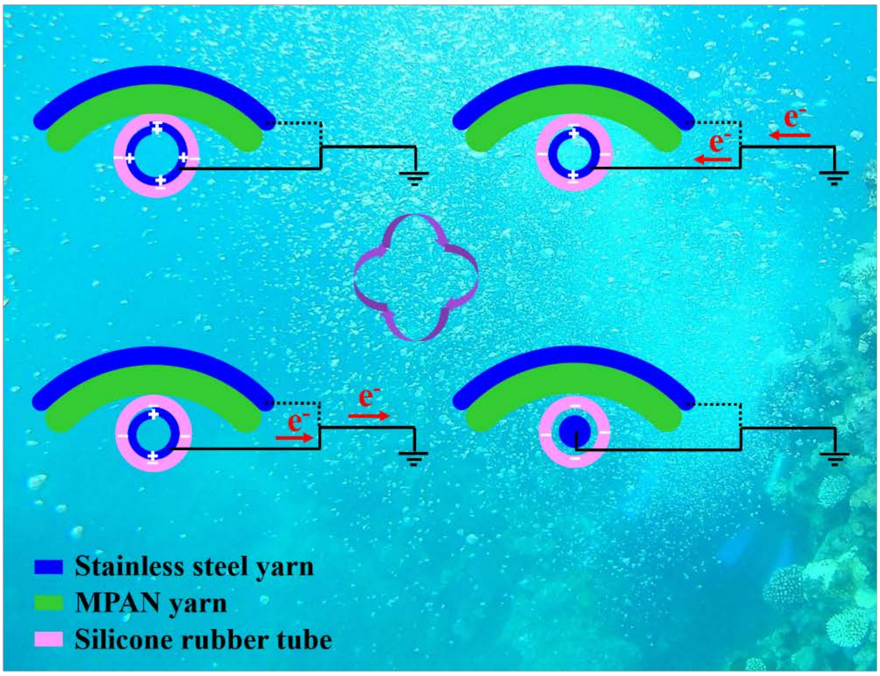

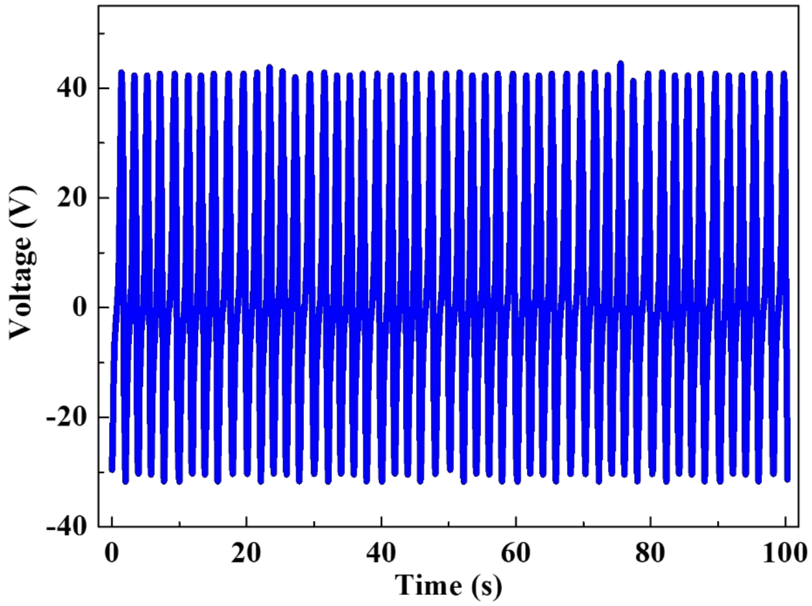

Supplementary Figure 38. (a) The working mechanism of the warp-weft-connection single-electrode e-textile when it is short-circuited in water. (b) The output voltage of the e-textile of the weft-connection single-electrode pattern underwater when the tensile strain is 100%.

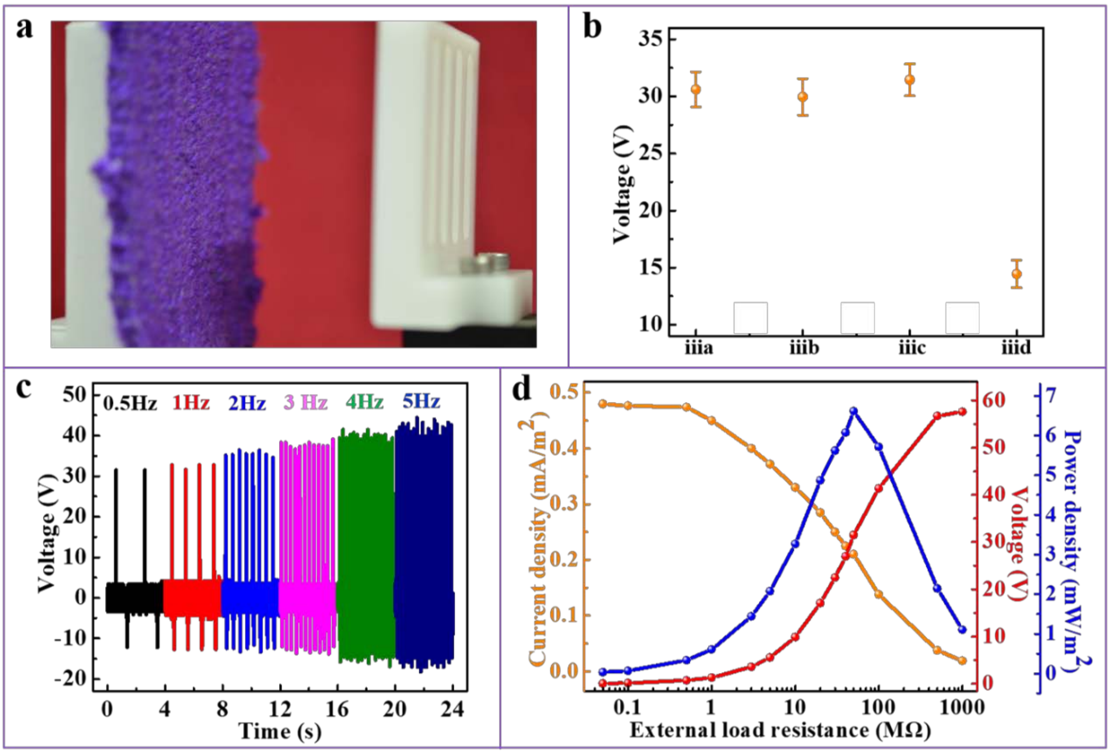

Supplementary Figure 39. (a) Photographs of the experiment setup with a contact-separation mode. (b) Output voltage of the four distinct patterns under compression at a fixed frequency of 0.5 Hz. The error bars correspond to standard deviation caused by the measurement noise. (c) Voltage of the warp-weft-connection single-electrode e-textile under compression at different frequency (0.5-5 Hz). (d) Electrical outputs of the warp-weft-connection single-electrode e-textile at different external load resistances with a tapping frequency of 0.5 Hz.

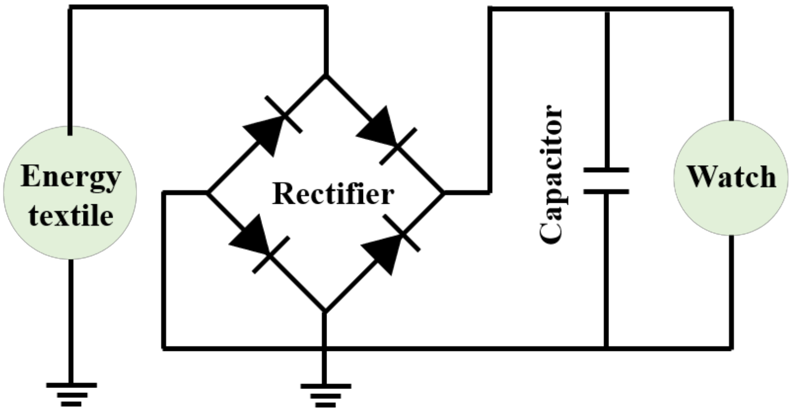

Supplementary Figure 40. The equivalent circuit of a self-charging system that uses the energy harvested from the e-textile to power an electronic watch.

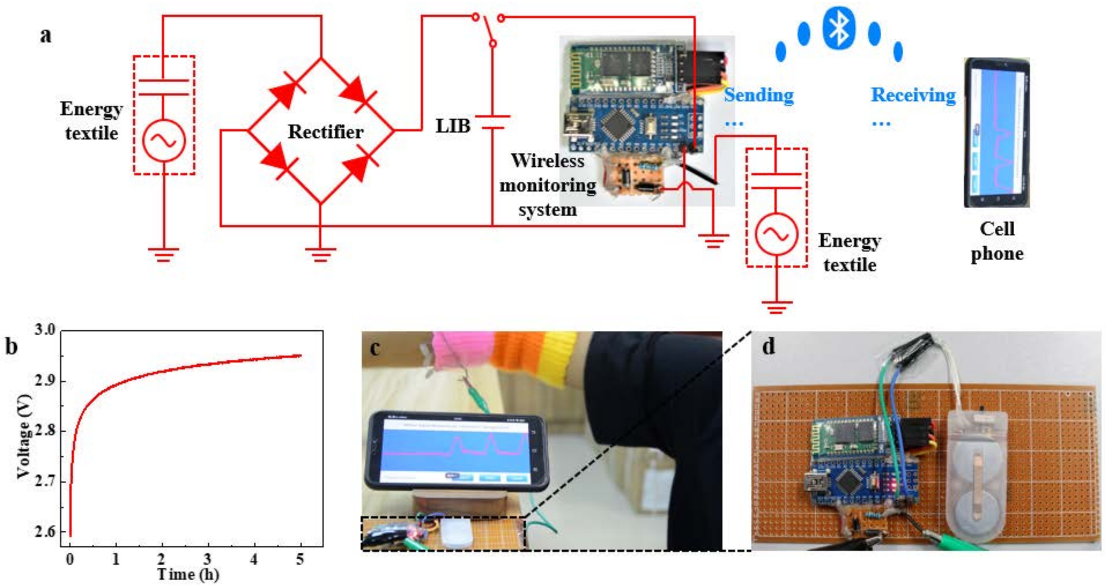

Supplementary Figure 41. (a) The equivalent circuit of a self-charging wireless monitoring system for real-time monitoring of body movements. (b) The charge curve of the Li-ion battery with the voltages ranged from 2.59 to 2.95 V. (c) A photo of real-time wireless monitoring of elbow bending. (d) A photo of the wireless monitoring system.

Supplementary References

1. Wang, Z. L. Triboelectric nanogenerators as new energy technology for self-powered systems and as active mechanical and chemical sensors. *ACS Nano* **7**, 9533-9557 (2013).
2. Saurenbach, F., Wollmann, D., Terris, B. D. & Diaz, A. F. Force Microscopy of Ion-Containing Polymer Surfaces: Morphology and Charge Structure. *Langmuir* **8**, 1199-1203 (1992).
3. Niu, S. et al. A theoretical study of grating structured triboelectric nanogenerators. *Energy Environ. Sci.* **7**, 2339-2349 (2014).
4. Yi, F. et al. Stretchable-rubber-based triboelectric nanogenerator and its application as self-powered body motion sensors. *Adv. Funct. Mater.* **25**, 3688-3696 (2015).
5. Niu, S. et al. Theoretical investigation and structural optimization of single-electrode triboelectric nanogenerators. *Adv. Funct. Mater.* **24**, 3332-3340 (2014).
6. Zhang, C., Gygi, F. & Galli, G. Strongly anisotropic dielectric relaxation of water at the nanoscale. *J. Phys. Chem. Lett.* **4**, 2477-2481 (2013).
7. Hill, N. E. Interpretation of the dielectric properties of water. T*ransactions of the Faraday Society* **59**, 344-346 (1963).
8. Fumagalli, L. et al. Anomalously low dielectric constant of confined water. *Science* **360**, 1339-1342 (2018).
