# Supplementary material for: Continuous and scalable manufacture of amphibious energy yarns and textiles
Source: Nat Commun. 2019 Feb 20;10:868. doi: 10.1038/s41467-019-08846-2 (PMC6382889; doi:10.1038/s41467-019-08846-2)
Supplement: Supplementary file 3 — Description of Additional Supplementary Files [file 41467_2019_8846_MOESM3_ESM.pdf]

Description of Additional Supplementary Files

File Name: Supplementary Movie 1
Description: This movie shows the process of pre-stretching the silicone rubber tube using the pulley blocks.

File Name: Supplementary Movie 2

Description: This movie shows the process of the SETEY collection by a reeling roller.

File Name: Supplementary Movie 3

Description: This movie shows the shape changes of the helical stainless steel yarn in a transparent silicone rubber tube.

File Name: Supplementary Movie 4

Description: This movie shows the self-powered application of the SETEY. The LCD is successfully lit up by the SETEY with hand motions in water.

File Name: Supplementary Movie 5

Description: The digital watch is successfully lighted up by the e-textile with arm movement (speed up by 1.5 times).

File Name: Supplementary Movie 6

Description: The LCD is successfully lit up by the e-textile with hand motions in water.

File Name: Supplementary Movie 7

Description: This movie shows a self-charging wireless monitoring system for real-time monitoring of body movements.
